# Machine Learning and Natural Language Processing in Mental Health: Systematic Review

**DOI:** 10.2196/15708

**Published:** 2021-05-04

**Authors:** Aziliz Le Glaz, Yannis Haralambous, Deok-Hee Kim-Dufor, Philippe Lenca, Romain Billot, Taylor C Ryan, Jonathan Marsh, Jordan DeVylder, Michel Walter, Sofian Berrouiguet, Christophe Lemey

**Affiliations:** 1 URCI Mental Health Department Brest Medical University Hospital Brest France; 2 IMT Atlantique, Lab-STICC, UMR CNRS 6285, F-29238 Brest France; 3 Department of Mental Health Johns Hopkins Bloomberg School of Public Health Baltimore, MD United States; 4 Fordham University Graduate School of Social Service New York, NY United States; 5 EA 7479 SPURBO Université de Bretagne Occidentale Brest France; 6 LaTIM, INSERM, UMR 1101 Brest France

**Keywords:** machine learning, natural language processing, artificial intelligence, data mining, mental health, psychiatry

## Abstract

**Background:**

Machine learning systems are part of the field of artificial intelligence that automatically learn models from data to make better decisions. Natural language processing (NLP), by using corpora and learning approaches, provides good performance in statistical tasks, such as text classification or sentiment mining.

**Objective:**

The primary aim of this systematic review was to summarize and characterize, in methodological and technical terms, studies that used machine learning and NLP techniques for mental health. The secondary aim was to consider the potential use of these methods in mental health clinical practice

**Methods:**

This systematic review follows the PRISMA (Preferred Reporting Items for Systematic Review and Meta-analysis) guidelines and is registered with PROSPERO (Prospective Register of Systematic Reviews; number CRD42019107376). The search was conducted using 4 medical databases (PubMed, Scopus, ScienceDirect, and PsycINFO) with the following keywords: machine learning, data mining, psychiatry, mental health, and mental disorder. The exclusion criteria were as follows: languages other than English, anonymization process, case studies, conference papers, and reviews. No limitations on publication dates were imposed.

**Results:**

A total of 327 articles were identified, of which 269 (82.3%) were excluded and 58 (17.7%) were included in the review. The results were organized through a qualitative perspective. Although studies had heterogeneous topics and methods, some themes emerged. Population studies could be grouped into 3 categories: patients included in medical databases, patients who came to the emergency room, and social media users. The main objectives were to extract symptoms, classify severity of illness, compare therapy effectiveness, provide psychopathological clues, and challenge the current nosography. Medical records and social media were the 2 major data sources. With regard to the methods used, preprocessing used the standard methods of NLP and unique identifier extraction dedicated to medical texts. Efficient classifiers were preferred rather than transparent functioning classifiers. Python was the most frequently used platform.

**Conclusions:**

Machine learning and NLP models have been highly topical issues in medicine in recent years and may be considered a new paradigm in medical research. However, these processes tend to confirm clinical hypotheses rather than developing entirely new information, and only one major category of the population (ie, social media users) is an imprecise cohort. Moreover, some language-specific features can improve the performance of NLP methods, and their extension to other languages should be more closely investigated. However, machine learning and NLP techniques provide useful information from unexplored data (ie, patients’ daily habits that are usually inaccessible to care providers). Before considering It as an additional tool of mental health care, ethical issues remain and should be discussed in a timely manner. Machine learning and NLP methods may offer multiple perspectives in mental health research but should also be considered as tools to support clinical practice.

## Introduction

### Machine Learning

Machine learning (ML) systems automatically learn models from data to make better decisions. As such, they are part of a major subfield of artificial intelligence (AI). There are 3 main approaches to learning from data: supervised, unsupervised, and reinforcement learning. In supervised learning, a target attribute is predicted, and ML algorithms infer a model from labeled input data (ie, a training data set that provides examples described by predictive attributes and values for the target attribute). The goal is to make target predictions on new data to obtain good generalization performance. In contrast, there is no target attribute in unsupervised learning, and thus no labeled data. Unsupervised learning consists of inferring a model to describe hidden patterns from unlabeled data. Under circumstances in which labeled data acquisition proves to be difficult, (eg, costly), semisupervised ML methods can use both labeled and unlabeled data for learning. The third main category of ML is reinforcement learning, in which the ML model uses feedback that acts as a reward or punishment to maximize its performance.

ML is limited to certain capacities. For one, it relies on collections of data that may be incomplete, noisy, or subject to systematic bias, all of which can lead to erroneous predictions. In addition, ML algorithms may introduce bias. Interesting questions to be addressed in ML are discussed in an article by Domingos [1]. However, when carefully conducted, ML can have great utility.

AI and ML have many applications, many of which are encountered in daily life. Supervised ML, for example, is widely used for spam filtering (ie, classifying incoming email as spam or not spam) [2]. It is also used to classify credit applicants based on their probabilities of default [3]. Unsupervised ML, such as algorithm clustering, is able to group customers with similar characteristics and their likelihood to purchase. This is widely used by banks for market segmentation [4]. Finally, automatic document clustering that organizes similar documents into classes (for purposes of improving information retrieval, for example) is gaining importance due to the increasing number of documents on the internet [5].

The application of ML in health is also of concern. Indeed, ML is widely used in critical disease models in cardiology, neurology, and diabetes research [6] to automatically identify heart disease risk factors [7], to classify primary progressive aphasia subtypes [8], and for the characterization and diagnosis of cognitive impairments [9], diabetes, and cardiovascular disorders [10-17].

ML is also challenging the traditional epidemiologic approach of evidence-based medicine owing to its high processing speed and ability to handle large volumes of data with heterogeneous variables (electronic health records, administrative data sets, wearable sensors, genomic and proteomic databanks, and social media) [18]. In fact, AI and ML have huge potential to build inferences and find patterns in vast volumes of patient histories, medical images, epidemiological statistics, and other particulars such as natural language data. For example, they can help doctors improve their diagnoses, forecast disease outbreaks, and customize treatments [19,20], provide better patient care [21], and predict the splicing activity of individual exons and chromatin marks from DNA sequences [22]. From a mental health perspective, the prevention of suicidal risk has recently been substantially studied [23-26].

Indeed, mental health care is also benefiting from the advancements in ML [27-29]. Classical ML with only mixed data (observations described by a mixture of numerical and categorical variables) is widely used, but language-based deficits are common symptoms of depression, bipolar disorder, autism spectrum disorder (ASD), personality disorder, and schizophrenia [30]. This implies that computational linguistics could have a great role in forming new insights into individuals’ mental health and emotions.

Language in both spoken and written forms plays an important role in ML mental health applications. It is therefore essential to understand what natural language processing (NLP) is before discussing the joint applications of ML and NLP in mental health.

### NLP

NLP is a subdiscipline of computer science that emerged in the 1960s. In 1967, the first published book on the subject, *Introduction to Computational Linguistics* [31], clearly considers language from a symbolic point of view: it describes techniques such as syntax parsing using dependency trees or Chomsky transformational grammars and statistical methods (word counting) are only hinted at. At that time, computing resources were sparse and had to be carefully managed; hence, a whole chapter of the book is dedicated to the storage of grammars in memory. The situation changed in the 1990s when personal computers became largely available and increasingly powerful. A new approach to NLP based on statistical methods emerged. The book by Manning and Schütze, *Foundations of Statistical Natural Language Process* [32], is a landmark of this evolution [32]. The 3 main sections of the book are dedicated to (1) methods at the word level (collocations, *n*-grams, and word sense disambiguation), (2) methods at the sentence level (morphosyntactic parsing using Markov models, and probabilistic context-free grammars), and (3) clustering, classification, and information retrieval. Probabilistic context-free grammars are a typical example of the evolution of NLP methods: the symbolic approach by Chomsky—or at least a simplified version—is endowed with probabilities attached to productions, and the ambiguity of natural language is reflected in the coexistence of several syntax trees with different probabilities.

During the same period, symbolic methods evolved as well. The 1990s witnessed the emergence of the World Wide Web, the Semantic Web, and ontology engineering. First, the 2 research directions seemed contradictory. Knowledge representation was aimed at structuring knowledge in an exhaustively precise symbolic manner, whereas the statistical viewpoint considered language in the same way as physics considers natural phenomena: by analyzing them through various heteroclitic methods, identifying general laws by numerical indicators, and proving them using statistical methods. An example illustrating the latter is the distributional semantic hypothesis (originally stated in the paper by Harris titled, Distributional structure [33]) asserting that “Words occurring in the same contexts will tend to have related meanings.” According to this hypothesis, one does not need to identify the precise meaning of a word, as a symbolic method would require, but simply to find the word’s cooccurrences in a corpus and consider these as semantics of the word. A very popular method called latent semantic analysis (LSA) is based on the following: the matrix of occurrences of words in documents (contexts) is reduced so that the dimensions of the new matrix represent aggregates of words and aggregates of documents where each dimension is not interpretable per se, but when words or documents are represented as vectors in this new *latent* system of coordinates, the scalar product of vectors can be used as a semantic relatedness measure [34]. LSA is also an example of a typical ML method, with a learning phase (when the frequencies of words in the corpus are counted and the word or document matrix is reduced) to perform a specific task (evaluating the similarity between documents).

Since the 2000s and 2010s, a new evolution has occurred in NLP with the emergence of convolutional, recurrent, and recursive neural networks (NNs) [35]. By using large corpora and sophisticated learning approaches, these methods provide good performance in tasks of statistical nature, such as text classification or sentiment mining. In the past 3 years, they have been much more frequently used for learning higher syntactic or semantic structures (syntax graphs or concept mining, respectively).

In the future, hybrid methods may be used more frequently, which combine symbolic and statistical approaches. The presence of ML methods in NLP systems is a trend that will undoubtedly remain integral to contemporary methods through the foreseeable future.

### Applications of ML and NLP to Mental Health

Applications of ML and NLP to mental health can be classified according to the following axes:

- The corpus: as one of the topics is NLP, the corpus necessarily has a textual component. The most common corpora are records or reports (electronic health records [EHRs], Psychological Evaluation Reports, and Coroner Reports), social media (Reddit, Twitter, etc), or transcribed patient interviews.
- Corpus processing: depending on the nature of the corpus, one can either extract medical terms and match them with unified medical language system (UMLS) concept unique identifiers (CUIs) or process blocks of text in natural language and perform specific searches (eg, to detect terms related to suicide).
- Classification methods: many ML techniques are used, such as decision trees, support vector machines, conditional random fields, random forests, and NNs.
- Goal: the goal is usually to validate a hypothesis or to study the behavior of a given population of patients.

Corpora can be of a very large volume. For instance, Sinnenberg et al [36] published a systematic review about Twitter as a tool for health research that included 137 different studies and analyzed over 5 billion tweets using ML; Castro et al [37] have processed 4.2 million EHRs spanning a period of over 20 years. Corpora can also be small, as demonstrated in the study conducted by Carson et al [38], who treated 73 respondents’ unstructured clinical notes, or in the study by Bedi et al [39], in which only 34 participants’ 1-hour-long narrative interviews were considered. Sometimes, corpora are created specifically for a project. For example, in a study by Roy et al [40], volunteers had written 182 abusive texts, annotated by researchers and abuse victims, and these texts were then analyzed and provided a model for detecting abusive texts.

Extraction of the UMLS CUIs is mainly applied to EHRs because the latter are semistructured and constitute a special document type. The specificities of this document type are reflected in its structure, the syntax of text, and, most importantly, the vocabulary used. The extraction of medical terms is achieved through information extraction algorithms and matching these terms with UMLS CUIs is performed through knowledge representation methods. Once these concepts have been extracted from an EHR, the latter is represented by the former and concepts become features used for classification.

On corpora other than EHRs, rather than extracting the UMLS CUIs, more general NLP methods are applied to textual data to obtain features that are then classified by ML algorithms. These NLP methods are often frequency counts of words or *n*-grams in a specific set, which can be manually curated or obtained out of a corpus. In other cases, methods such as LSA or latent Dirichlet allocation (LDA) are used for topic detection. The initial set of words can be explicit. For example, Doan et al [41] collected tweets containing the hashtags #relax and #stressed and classified them by theme and location. In other cases, calculations are performed at a higher level and words involved in the process are not explicitly known. For example, Luo et al [42] attempted to characterize autism by analyzing textual descriptions of closely related individuals written by patients or members of a control group. Nevertheless, most NLP applications in mental health rely on words (using the bag-of-words method, that is, ignoring word order and keeping only their frequencies). Some take word order into account in a limited way (by using *n*-grams, ie, contiguous sequences of words of length *n*), but very few take syntax into account by the use of dependency trees [18,43,44]. With respect to their applications, it should be noted that ML and NLP tools are invaluable in alleviating data issues such as data overflow in modern medicine. Forsting et al [45] acknowledge that ML and NLP techniques can be useful for optimism bias (eg, the difference between a person’s expectation and the actual outcome or the concept that a clinician may think that his or her patient’s problem falls solely into a specific discipline in which the physician works) because the machine has a generalist approach unlike the specialist clinician. Within the last two decades, these techniques have emerged in mental health, following the success of social media to act as an informative source of data [46].

In addition, NLP is essential in psychiatry because language-based deficits are common symptoms of depression, behavioral disorder, ASD, personality disorder, and schizophrenia [30]. It can provide insight into individuals’ mental health and emotions, their use of narrative, subjective, and structured speech styles, and their lifestyle, specifically their educational level, socioeconomic status, living conditions, and cultural background [47], all of which are routine in mental status examinations.

Using ML in general and NLP methods in particular, one can create semiautomated systems (operating under human supervision) aiming to improve the specificity of diagnosis, knowledge of psychophysiology, speed of diagnosis, and more accurate estimations of disease severity [48]. Through analyses of Twitter posts, O’Dea et al [49] identified the importance of creating real-time campaigns to increase help-seeking behaviors and reduce the stigma attached to mental health. Moreover, automated programs can be more cost-effective and time-efficient than their traditional counterparts. Ly et al [50] proposed using interventions based on an automated self-help system as a way to make mental health promotion tools more widely accessible. In addition, Lucas et al [51] demonstrated through a clinical trial that when people believed they were interacting with a computer rather than an actual clinician, they reported less fear of self-disclosure, reported reduced impression management behaviors, experienced more ease in expressing the severity of their emotions, and were rated by observers as more willing to disclose. However, these findings may not be generalizable, as they were potentially biased by their sample selections and/or system design itself.

Although ML and NLP provide new tools and strategies for psychiatric research and practice [52], it should be kept in mind that their use frequently raises ethical and legal concerns over consent to personal data use and data anonymization. Similarly, studies using AI for predictive analyses are challenging the balance between beneficence and respect for patients’ autonomy. McKernan et al [53] suggest that efforts be made to communicate AI methods to obtain free and informed consent from patients. Moreover, prospective studies should be conducted to evaluate the use of AI tools [53].

The primary aim of this systematic review is to summarize and characterize studies that used ML and NLP techniques for mental health in methodological and technical terms. Hence, the secondary aim is to consider the potential use of these methods in mental health clinical practice, such as the contributions that they may offer in areas of diagnosis and prognosis, the establishment of risk factors, impacts of psychotherapy, treatment adherence, and side effects.

## Methods

This systematic review is grounded in the PRISMA (Preferred Reporting Items for Systematic Reviews and Meta-analysis) guidelines [54]. Searches were carried out as specified by the standard protocol for PROSPERO (Prospective Register of Systematic Reviews; registration number CRD42019107376).

### Literature Search Strategy

A systematic, computerized literature search was conducted using 4 databases: PubMed (via MEDLINE), Scopus, ScienceDirect, and PsycINFO. Each database was explored from August 21, 2018, through February 1, 2020, with no publication date limit. The search was carried out using the following keywords: “natural language processing” AND “machine learning” AND (“psychiatry” OR “mental health” OR “mental disorder”). The same search was performed on the element (data mining) instead of (machine learning). When the full text was not available, the abstract was used to extract the necessary information to avoid selection bias. Case studies, conference papers, and reviews were excluded.

### Study Selection and Eligibility Criteria

After removing duplicates, 2 collaborators independently screened all titles and abstracts that were relevant to this systematic review. A third reviewer was consulted when disagreement arose between the first 2 collaborators. The process is depicted in Multimedia Appendix 1. Only studies available in English were selected. We deliberately excluded studies about the anonymization process to focus on the articles investigating the clinical use of ML and NLP in psychiatry (eg, contribution to diagnosis, prognosis, establishment of risk factors, impact of psychotherapy, treatment adherence, and side effect). No limitations on publication dates were imposed. A total of 58 articles were included in the review.

### Included Studies

All studies were thoroughly screened, and their main ideas are summarized in individual tables (Multimedia Appendix 2 [37-41,43,47,48,55-104]). These tables provide information on qualitative and quantitative features: authors, year of publication, precise topic of mental health (eg, autism, psychotic spectrum disorder, etc), population characteristics, and types and volume of recorded data. The second part of these tables summarizes the objectives, methods, and results.

## Results

### Study Selection

The database search resulted in 222 studies identified using the (machine learning) keyword and 105 studies using the (data mining) keyword. After merging them, 238 unique studies were considered for review, based on the title and abstract. A total of 84 papers were excluded because (1) they were not about psychiatry or mental health (52 cases), (2) they were not written in English (1 case), and (3) the keywords (machine learning), (natural language processing), or (data mining) did not appear in the title or abstract (8 cases). As a second filter, 33 studies about data anonymization were excluded. Furthermore, 7 studies were excluded because ML or NLP were not their main subject but were only quoted as background information. In addition, 96 papers were excluded because they were reviews, case studies, or conference papers. Finally, 58 articles were included in this review.

### Topics and Population

Topics are heterogeneous. The most frequently mentioned topics are depression and suicide with 17 studies [38,55,57,60-62,77-79,82,83,87,88,91,92,99,104]. Other psychiatric diagnoses were addiction to alcohol or illicit drugs (6 cases) [43,65,66,75,84,86]; posttraumatic stress disorder (PTSD; 3 cases) [47,63,64]; neurodevelopmental disorders (3 cases) [42,58,93]; psychotic spectrum disorders, including schizophrenia (3 cases) [39,95,100]; anxiety (2 cases) [41,98]; personality disorder (1 case) [85]; eating disorders (2 cases) [89,96]; and bipolar disorder (2 cases) [37,102]. A total of 3 studies were on violence and cyber harassment [40,80,94]. Treatment issues such as adherence or misuse are also depicted (6 cases) [56,72,74,81,101,103]. Only 1 study on mechanical restraints [90] and 1 on cognitive troubles [97] were found. A total of 8 studies were transnosographic [59,67-71,73,76]: 6 met the CEGS N-GRID 2016 Center of Excellence in Genomic Science Neuropsychiatric-Genome-Scale and Research Domain Criteria (RDoC) Individualized Domains 2016 Shared Task in Clinical NLP criteria, which will be developed further in our results.

In total, 3 distinct categories of population were found:

1. Patients whose EHRs were available in science-based research databases such as the Partners HealthCare electronic medical record (EMR), a collection of data from patients at Massachusetts General Hospital and Brigham and Women’s Hospital [55,56]. These records extended beyond psychiatric records and included other medical records as well.
2. Patients seen in emergency or psychiatry departments who had additional clinical characteristics in their records (eg, clinical observation, laboratory tests, diagnostic and therapeutic interventions, typed specialists’ notes).
3. Social media networks (Facebook, Twitter, and Instagram): The authors of these studies have selected specific hashtags such as #stress or #depression and have screened a multitude of public messages using a streaming platform.

#### Objectives

In total, 5 main categories of objectives were found: to extract clinical symptoms, to classify severity of illnesses, to compare different therapies, to provide psychopathological clues in mental health, and to challenge the current nosography.

The principal objectives of these studies were to extract and record clinical symptoms, establish a diagnosis, or monitor changes over time. A total of 2 studies targeted automated epidemiological monitoring: Metzger et al [57] provided a method of detecting suicide attempts from EHRs and Leroy et al [58] achieved automatic extraction of criteria for ASD from EHRs with an accuracy of 76%. The latter study stated that an increasing prevalence of given symptoms (nonverbal behavior, social and emotional reciprocity, and adherence to routine disabilities) occurred from 2000 through 2010. Data extraction was also used for diagnosis: He et al [47] diagnosed PTSD with an accuracy of 82% after analyzing free texts written by trauma survivors.

In addition to extraction, an important aim was to measure the severity of psychiatric disorders in psychological evaluation record corpora. Goodwin et al [59] classified symptoms of patients with psychosis into 4 different levels of severity (absent, mild, moderate, and severe) using statistical analyses. Fernandes et al [60] studied EHRs from a cohort of individuals with a history of suicide attempts and a cohort of individuals with a history of suicidal ideation only. Their algorithm of detecting suicidal ideation or suicide attempts had a sensitivity of 98.2% and a positive predictive value of 82.8% [57]. Other studies found that ML and NLP techniques performed well, although they were not necessarily better than a practitioner’s ability to predict the clinical risk of suicide in their patients [61,62]; thus, the authors proposed statistical NLP approaches to be used in collaboration with clinical practice.

ML and NLP methods are also used to measure and compare the effectiveness of different types of psychotherapy [63,64]. Tanana et al [43] investigated 2 statistical NLP techniques to code motivational interviewing sessions. Motivational interviewing is a psychotherapy method used for substance use disorders and other behavioral problems to strengthen personal motivation for change [105]. Motivational interviews can be manually coded to assess therapy adherence and gather feedback for subsequent sessions. The authors found that the discrete sentence feature model (a sentence classifier based on *n*-gram models) had accuracy similar to the manual coding of therapeutic sessions. Maguen et al [63] used statistical NLP techniques to distinguish evidence-based psychotherapy, including cognitive processing therapy and prolonged exposure notes from unstructured psychotherapy notes for a population of veterans with PTSD. They found that almost 20% of veterans observed an improvement in their symptoms after one or more sessions of evidence-based psychotherapy.

Another objective was to provide psychopathological clues for understanding mental health disorders by analyzing language features. This objective sometimes involves the processing of previously unexplored data, such as chat groups or social networks. The following are some examples of studies that pursue this objective: Baggott et al [65] found that MDMA (3,4-méthylènedioxy-N-méthylamphétamine; Ecstasy) altered individuals’ speech patterns more frequently than the placebo and led to an increase in both positive and negative social and sexual language use (others, public, camaraderie, and outgoing). Chary et al [66] analyzed posts on Lycaeum, a popular web forum known for being one of the most frequently cited platforms with respect to drug use. They discovered new combinations of drugs that were not mentioned in the medical literature. Luo et al [42] differentiated the social interactions between adults with ASD and healthy adults. They confirmed the hypothesis regarding differences in language and social interactions in adults with ASD: typical participants had more connected semantic links than the ASD group and the words with the largest number of connections were different between the 2 groups. Doan et al [41] noticed that American Twitter users are more likely to express their source of stress on Twitter than in their day-to-day experiences. The main causes of stress that emerged from the Twitter data were education, work, and social relationships. They also found that individuals’ expressions of stress and relaxation differed based on the city of residence (Los Angeles, New York, San Diego, and San Francisco). Moreover, Mowery et al [106] revealed that less than 2% of the tweet corpus (a corpus of 9300 annotated tweets containing depression-related keywords) included more than one depression-related reference, suggesting that there may be different forms of expression when it comes to depression.

Finally, AI in mental health research challenges the current practice and nosography. In 2010, Insel et al [107] initiated a project called the RDoC, a research framework for mental health disorders that aims to constitute an alternative to the DSM (Diagnostic and Statistical Manual of Mental Disorders). The former includes data on genetics and neuroscience in its classification of mental health disorders, whereas the latter is solely based on clinical data [107]. The RDoC is a matrix in which the columns and rows represent constructs (genes, molecules, cells, circuits, physiology, behaviors, self-reports, and paradigms) and subconstructs of each of the following 6 domains: negative valence, positive valence, cognitive systems, systems for social processes, arousal or regulatory systems, and sensorimotor systems. Pro-RDoC practitioners argue that DSM syndromes have significant limitations when used as phenotypes for identifying biomarkers and specific genetic variants associated with mental illness [108]. A concrete application of this new system used statistical NLP methods to create a phenotypic homogenous cohort that allowed a better comparison [109]. In 2016, the CEGS N-GRID (Centers of Excellence in Genomic Science Neuropsychiatric-Genome-Scale and RDoC Individualized Domains) proposed 3 challenging tasks using NLP methods: (1) data anonymization, (2) predicting symptom severity in the positive valence domain from neuropsychiatric clinical records, and (3) novel data use case (eg, predicting the presence of common mental conditions in patients) [67]. This research on NLP and ML processing identified 6 articles [59,67-71] that met these challenge tasks, although only 1 study dealt with task 3 [67]. As mentioned earlier, studies on anonymization were excluded; thus, the RDoC framework links the neuro-biological basis of mental processes with phenotypical manifestations [110]. The CEGS N-GRID shared task provided usable data for investigating ML and NLP techniques, which could lead to new psychiatric nosology.

#### Type of Data Used

As can be seen in Table 1 (in which no hapaxes are displayed), the most frequent corpus type is that of EHRs (to which EMRs can be added). EHRs (and EMRs) are convenient data sources because of their heterogeneity: they combine structured, semistructured, and free data, and they often use a significantly controlled language containing medical terms that allow the extraction of CUIs (Methods Section). The second most frequent sources of data are clinical notes and clinical records, which share the convenient properties of EHRs or EMRs, but are not standardized in the same way.

**Table 1:** Corpus type.

Characteristics	Values
EHRs^a^	22.9508
ClinNotes	16.3934
ClinRecords	11.4754
Interviews	8.1967
Tweets	8.1967
Questionnaires	6.5574
Reddit	6.5574
Web	4.918
EMRs^b^	3.2787

^a^EHR: electronic health record.

^b^EMR: electronic medical record.

The data described earlier share an important property: the corpora are generated by practitioners and therefore can be used for medical term extraction with satisfactory results.

A different category of data is generated by the patients. This category can be divided into 2 subcategories: data generated with the help of practitioners (eg, interviews and questionnaires) and data freely generated by patients on social media (tweets, posts on Reddit, web blogs, etc).

Interviews and (textual parts of) questionnaires are technically free text but practitioners still have some amount of control over the content, and the environment in which the data are collected influences the degree of informality of texts. For these reasons, traditional NLP methods can be applied to them with satisfactory results.

Data collected from social media, because of their high degree of informality, loose spelling and syntax, and use of abbreviations and emojis, can only be superficially processed by standard NLP methods. Typical examples are in studies by Doan et al [41] and Jackson et al [73], in which tweets were selected because they contained the hashtags #stress and #relax and their words were used in a bag-of-words without any further linguistic treatment [41] or tweets were selected based on the presence of terms denoting opioids [72]. Although the authors lemmatized tweet contents, the main feature of tweets taken into account was their geographical origin.

#### Methodology

Two phases of NLP projects were distinguished: (1) preprocessing, which consists of analyzing the data to obtain numeric or categorical features, and (2) classification.

##### Preprocessing

Table 2 (in which no hapaxes are displayed) represents the frequency of use of various preprocessing methods that can be of different natures. Some methods apply to words or word groups: *lemma (*lemmatization, ie, replacing a word by a base form such as the singular for nouns or the infinitive for verbs), *POS* (part of speech, ie, attaching to a word a label denoting its grammatical function), *cTAKES* or *CUIs* (mapping a word or a noun phrase to concept in an ontology, such as the UMLS, and therefore unambiguously defining its semantics), *tf-idf* (attaching to a word or a term a value representing its significance in characterizing a given document or class it belongs to), *embedding* (representing a word by a vector in a high-dimensional space), *named-entity recognition* (deciding whether a given word or noun phrase is a named entity), *LIWC* (Linguistic Inquiry and Word Count, a commercial tool advertised as being “based on solid science” providing various “social and psychological insights” of words). Other methods combine words into higher structures: *n-grams* (considering an *n*-gram, ie, a sequence of *n* subsequent words, as an entity and measuring the frequencies of these entities). Finally, other methods are applied to entire sentences, paragraphs, or documents: *SentiAna* (analyzing sentiments or emotions), *LDA* and *LSA* (calculating sets of topics, detecting the significance of each topic for a given document, and providing representative words for each topic). The most frequent preprocessing methods are the standard methods of NLP (lemmatization, part-of-speech tagging, *n*-grams, and tf-idf), and methods specific to medical texts such as CUI extraction (keywords cTAKES and CUIs in Table 2). The embedding method is related almost exclusively to NNs and therefore is relatively recent. Finally, the tail of the graph in Table 2 contains methods applied primarily to free texts such as topic detection, named-entity recognition, sentiment or emotion analysis.

**Table 2:** Preprocessing methods.

Characteristics	Values
lemma	16.3043
POS^a^	10.8696
cTAKES^b^	10.8696
ngrams	9.7826
tfidf	7.6087
embedding	6.5217
CUIs^c^	5.4348
LDA^d^	5.4348
SentiAna	5.4348
LIWC^e^	4.3478
NER^f^	4.3478
LSA^g^	3.2609

^a^POS: part of speech.

^b^cTAKES: clinical Text Analysis and Knowledge Extraction System.

^c^CUI: concept unique identifier.

^d^LDA: latent Dirichlet allocation.

^e^LIWC: Linguistic Inquiry and Word Count.

^f^NER: named-entity recognition

^g^LSA: latent semantic analysis.

##### Classification

Once the classification phase is reached, linguistic data are entirely converted into numeric data, and therefore, the choice of classifier depends on factors other than corpus type. Some of these factors include (1) the volume of data, (2) the type of classification (supervised vs unsupervised), (3) the explicability level, and (4) the platform used. In Table 3 (where hapaxes are not displayed), we have shown decision tree, association rules, and C4.5 (also a decision tree algorithm) that are *transparent* methods, that is, the user can follow the classification process in a step-by-step manner and understand the reason a given individual belongs to a particular class. They are not the most frequent classifiers, probably because explicability is not a major concern of most studies. Instead, the most frequently used classifiers such as support vector machine (SVM), LogiR (logistic regression), RF (random forest), and LinR (linear regression) are solid, fast legacy classifiers with small parameter sets and good performance. In the middle of Table 3 are NNs that belong to the deep learning tendency of ML: they are opposite to DT/AR/C4.5 when it comes to explicability and they rely heavily on certain parameters (type and geometry of NN, number of layers, size of layers, optimizer, learning rate, loss function, etc). The causes of the relatively low frequency of NNs in publications may be (1) the fact that they have been implemented in user-friendly frameworks (such as Theano or Keras) only recently, (2) the necessity to fine-tune a large number of parameters, and (3) the relatively high requirements in terms of memory, central processing unit, and graphical processing unit. This is likely to change in the near future.

**Table 3:** Classifier type.

Characteristics	Values
SVM^a^	22.6804
LogiR^b^	16.4948
RF^c^	11.3402
DT^d^	6.1856
NB^e^	6.1856
NN^f^	6.1856
LinR^g^	5.1546
K-Means	3.0928
AR^h^	2.0619
C4.5	2.0619

^a^SVM: support vector machine.

^b^LogiR: logistic regression.

^c^RF: random forest.

^d^DT: decision tree.

^e^NB: Naive Bayes.

^f^NN: neural network.

^g^LinR: linear regression.

^h^AR: association rules.

##### Platforms

As can be seen in Table 4 (hapaxes are not represented), the 2 most common platforms are Python and R. Python is a *universal* programming language, in the sense that it is not specific to a given domain: more than 120,000 packages allow the user to perform specialized tasks in any possible field. Furthermore, it is open-source and high-quality documentation abounds. R is also an open-source programming language and compiler, but contrary to Python, it is oriented toward statistics. Although many classifiers have been implemented efficiently both in Python and R, the domain of NLP is better represented in Python, in credit to packages such as NLTK (Natural Language ToolKit), spaCy, and Stanza. The third bar, titled *Unknown*, represents publications that do not mention the platform used. The fourth bar indicates the General Architecture for Text Engineering General Health platform, an open-source Java application that provides an environment for processing textual data in a user-friendly manner. The *Apache* bar gathers different tools distributed by the Apache Software Foundation. Stata is a commercial statistics software from College Station, Texas, first released in 1985. Weka is an open-source programming environment for ML.

**Figure 1:**
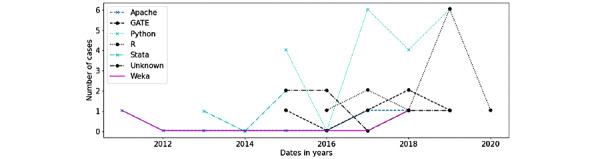
Platforms usage.

Figure 1 shows the use of platforms in chronological order. The use of Python and R started after 2015, while Stata, Weka, and Apache were already in use in 2011.

**Table 4:** Platforms.

Characteristics	Values
Python	34.4828
R	18.9655
Unknown	10.3448
GATE^a^	8.6207
Apache	5.1724
Stata	5.1724
Weka	3.4483

^a^GATE: General Architecture for Text Engineering General Health.

### Correspondence Analysis of Data, Methods, Classifiers, Platforms, and Publications

**Figure 2:**
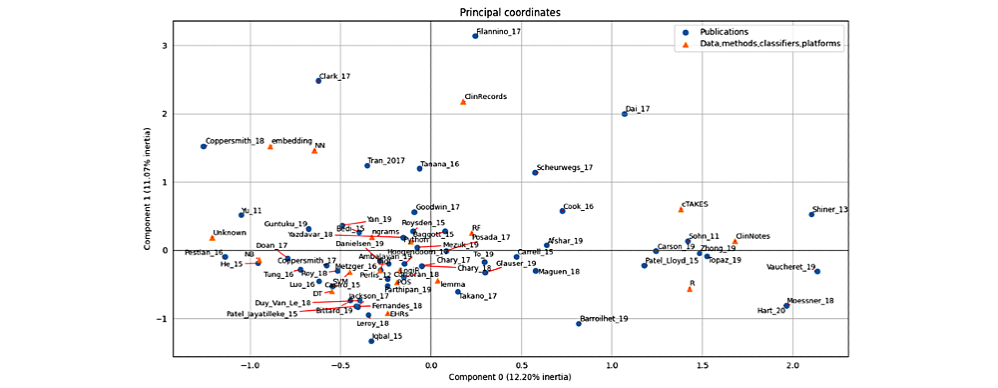
Correspondence analysis.

The correspondence analysis is a dimension reduction technique that maps the data into a factorial space where each dimension is a combination of the initial variables. Figure 2 represents the principal coordinates of the publications and the various entities considered in their study.

On the right, a cluster of publications is surrounded by data type *ClinNotes*, method *cTAKES*, and platform *R*. In the upper left quadrant, some publications gather with method *embedding* and classifier *NN*. Toward the left of the diagram and close to the horizontal axis, publications with an *unknown* platform using the *NB* classifier are present along with a big cluster whose center includes *tf-idf*, *LogiR*, *SVM*, *Python*, and *n-grams*: the legacy methods, most used classifiers, and the most used platform.

With regard to publications, *Filannino_17* is an obvious outlier because it has no method, classifier, or platform and because it describes a task and how this task has been treated by others. *Clark_17* is at the extreme upper left, as it uses NNs and k-means (the latter is not displayed because only entities appearing at least 5 times are included). *Coppersmith_18* also uses embeddings and NNs, whereas *Tran_17* (which is closer to the central cluster) uses both NNs and SVMs. On the right side, *Shiner_13* and *Vaucheret_19* use clinical notes and R, whereas *Hart_20* and *Moessner_18* use R and methods that have not been taken into account in the calculation. In the bottom left, *Iqbal_15* uses EHRs in the General Architecture for Text Engineering General Health (which is not displayed). At the extreme left and close to the horizontal axis, *Pestian_16* and *Yu_11* use an unknown platform.

### Geographical Distribution of Authors

**Figure 3:**
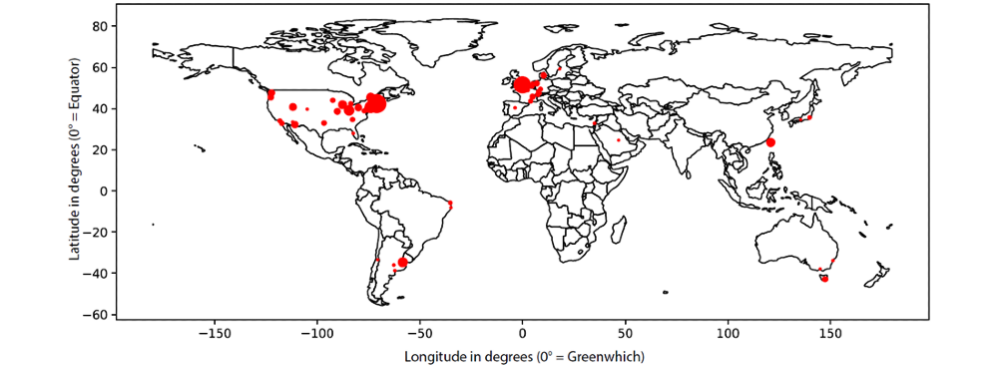
Geographical distribution of authors.

In the map in Figure 3, the diameter of the red marks is proportional to a score calculated as follows: we added 1 unit for the geographical origin of the affiliation of each author of each paper. The cities with scores greater than 10 were Boston (54), London (44), New York (21), Cincinnati (15), Buenos Aires (13), Cambridge, Massachusetts (12), San Francisco (11), and Taiwan (11).

### Citations and Cocitations

**Figure 4:**
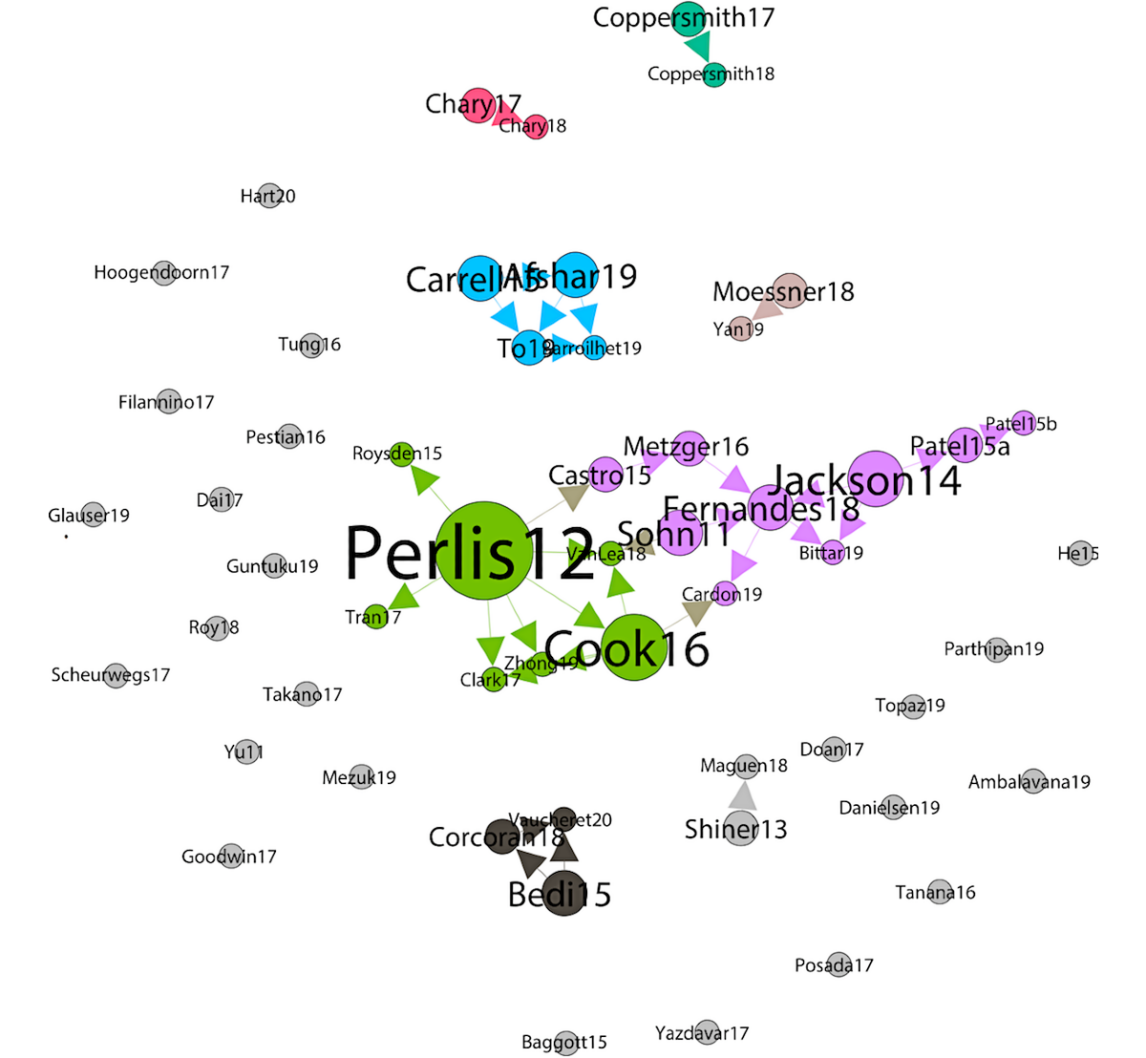
Graph of cocitations.

Figure 4 represents the citations of the papers in our list by other papers on the same list. The size of the nodes of a paper is proportional to the number of papers citing it. The colors of the nodes and edges represent communities. Each community has a central node: Perlis et al [55] are cited in 7 other papers, Jackson et al [73] are cited in 4 other papers, Carrell et al [74] and Afshar et al [75] are cited in 2 other papers, and Bedi et al [39] are cited in 2 other papers. In total, 22 papers are singletons: they are neither cited nor cite any other paper in our list.

**Figure 5:**
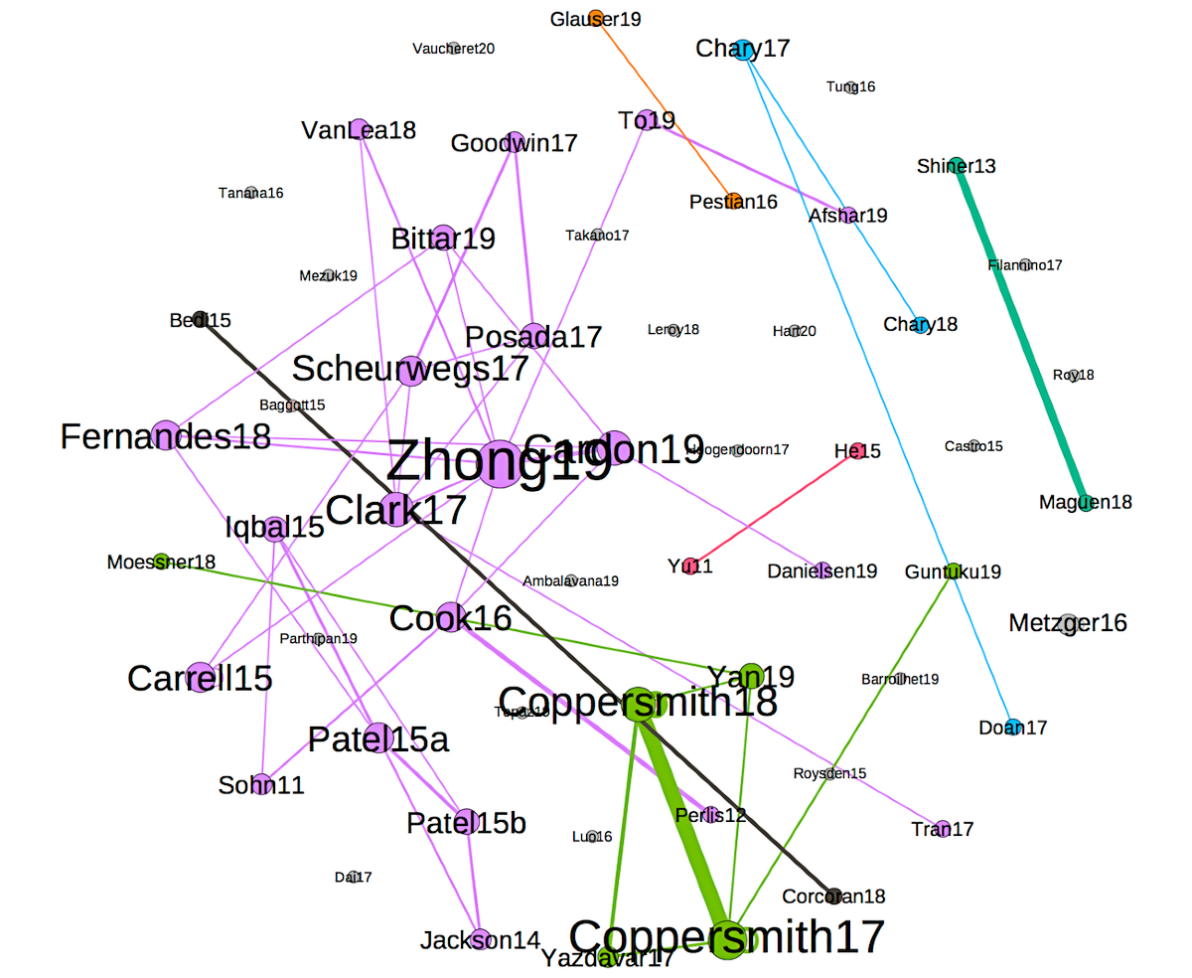
Graph of cocitations.

Although mutual citations show influences between papers in our list, we can also measure the number of cocitations (ie, common references between 2 papers in the list). In Figure 5, the edges between papers indicate that they have at least 3 common references. The edge width is proportional to the number of references.

The edge of the greatest width is the one between the papers by Coppersmith et al [76] and Coppersmith et al [77], which is normal—the 2 papers share the same first author, have been released within less than a year, and have 26 common references.

The second case, in descending order of edge width, is between Shiner et al [64] and Maguen et al [63]. This is also normal—the first author of the former is also the last author of the latter and the latter is presented as an extension of the former: “In this study, our goal was to extend Shiner and colleagues’ work by applying automated coding to a large national pool of mental health treatment notes in order to identify the use of cognitive processing therapy and prolonged exposure.” The 2 papers share 14 common references.

The size of nodes in the graph is proportional to the degree. Zhong et al [78] have the highest degree: this paper has more than three common references with as many as eight other papers, in fact, with 8 references. The color of the nodes and edges corresponds to the connected components. There are 19 singleton nodes that share ≤2 references with every other paper of the list.

## Discussion

### Strengths and Limitations of the Review

This study reviews ML and NLP models in the field of mental health, which has been a highly topical issue in recent years. The methodology was elaborated to screen a maximum number of specific medical studies by expanding the research to 4 medical databases (PubMed, Scopus, ScienceDirect, and PsycINFO). Furthermore, the characterization of the selected studies has been done very precisely in a qualitative manner to simultaneously depict the populations, methods, data sources, and technical aspects.

The primary limitation of this study is the lack of quantitative comparisons between the selected studies. It is indeed not feasible to compare highly heterogeneous studies that do not share common research patterns. In addition, the selected works were not scored on their risk of bias. Despite this shortcoming, their limitations and strengths are outlined in the individual tables in Multimedia Appendix 2.

#### Methodological and Technical Limitations of the Selected Studies

ML and NLP methods may be considered as a new paradigm in medical research in which it becomes practical to analyze every possible, even unexpected, and innovative parameter of a topic to discern new clinical patterns. This new paradigm involves reconsidering the standard methodology, which consists of formulating a sound hypothesis, defining objectives, and collecting results to either uphold or reject the hypothesis. However, in practice, the selected studies tend to confirm clinical hypotheses based on fundamental clinical intuitions, namely language abnormalities in adults with ASD [42].

Other methodological limitations and potential bias sources have been noted. As stated in the Results section, one of the 3 main population categories is *social network or chat users* [40,41,66,77,79], whose members are predominantly young. Owing to this, Coppersmith et al [76,77] cautioned that these results may not be generalizable to other populations [77,106]. In addition, when Chary et al [66] focused on *Lycaeum users* and Coppersmith et al [76] mentioned *participants from a company*, the lack of precise information on the participants of a cohort was obvious. An exception to this is the group of OurDataHelps.org users [77] who volunteered to participate in scientific research and filled out a questionnaire to provide information about themselves. Even when participants volunteer to provide personal information, there is a high likelihood that personality bias plays a role, especially in studies on suicide and depression.

Similarly, studies rarely consider cultural or ethnic differences within a sample [80]. For example, in a study on violent behavior, researchers should acknowledge that *spanking children for discipline purposes* is considered inappropriate in some cultures but appropriate in others. In some cases, language-specific features can improve the performance of NLP methods. For example, in the case of Takano et al [62], the distribution of morphemes is used to distinguish between specific and nonspecific memories in the Autobiographical Memory Test. As shown in the paper, among the most important distinctive factors are grammatical particles that are specific to the Japanese language, such as た/だ (past tense), ない (negation), は (topic marker), and で (place or method). In languages with different structures, the same method may be less efficient and other indicators may need to be investigated.

#### Is There an Advantage in Using ML and NLP for Mental Health Clinical Practice?

The hallmark ML principle is to simultaneously analyze large quantities of data; however, this sometimes leads researchers to the implicit assumption that *the more data they input, the more accurate will be the results*. ML and NL allow the analysis of large amounts of data and the comparison of broad groups and patients. For example, Roysden et al [56] screened administrative data and EHRs from a population of 12,759 patients; Maguen et al [63] compared over 8,168,330 clinical notes collected over 15 years; and Yazdavar et al [79] analyzed posts authored by 4000 Twitter users. At the same time, even though thousands of papers have been published using medical data, very few have made meaningful contributions to clinical practices [111].

Twitter and other social networks, with almost 3 billion users globally, have become significant sources of information for medical use and research [112]. Moreover, the analysis of social media–based platforms can generate valuable details about people’s mental health and social or professional interactions. The alteration of daily habits is one of the core criteria for the diagnosis of a mental health disorder (in general, criterion B of DSM-5). A recent study by Fagherazzi and Ravaud [113] illustrates the idea that AI can be implemented in the so-called *digitosome* (data generated online and by digital technologies) that constitutes a powerful agent for detecting new digital markers and risk factors in medicine. By analyzing a global cohort of more than 85,000 tweets per week authored by people with diabetes, they were able to discuss different illness-related stress patterns of patients with type 1 or type 2 diabetes. By analyzing tweets, Mowery et al [106] found that there may be alternative ways in which people express depression. These findings indicate that there may be new ways for people to express mental illness.

From this perspective, different expressions of psychological distress (whether people are addressing health care professionals, relatives, or digital friend networks) could be accessible and useful to care providers. ML and NLP may be valuable in psychiatry for identifying people with clinical risks for depression, suicide attempts, anxiety, or even psychosis based on digital data or clinical notes.

#### Ethical Reflections

AI in psychiatry and more broadly in medicine raises ethical issues and requires prudence in its application. As mentioned earlier, ML and NLP techniques have valuable advantages in psychiatry for analyzing large amounts of data with high diagnostic and prognostic validity. These tools, which have been groundbreaking in medicine and psychiatry, should receive more attention for their promising results with regard to clinical practice and medical research. In addition, recent studies suggest that people are becoming more comfortable when speaking with a machine compared with a clinician: Lucas et al [51] state that in a clinical trial, people who (believed they) were interacting with a computer disclosed information more openly than people who thought that an individual was controlling the computer. Perhaps the machine is viewed as being more objective than a human and therefore reduces the fear of judgment from a practitioner. The introduction of a computer in medical practice as a new type of clinician leads to a profound change in the physician-patient relationship and promotes the idea of having a new clinical model involving a third party. The relationship is crucial to psychiatric clinical practice, and the use of data processing should be discussed. Sassolas [114] questioned this *technological psychiatry* as a practice that is likely to avoid what he called the “psychic privacy proximity.” Technological psychiatry could generate an operative encounter whose unique purpose is to normalize the patient’s symptoms and reduce the fear of disclosure.

In addition to improved relationships, the application of ML and NLP in psychiatry should be done with special precautions to avoid clinical abuse. This review includes 2 studies about the prediction of psychosis in patients at high risk of this disease. One even introduced a model of ML+NLP that had a 100% accuracy in predicting psychosis among the latter patient sample [39], which was better than a simple clinical evaluation. Nevertheless, these results should be treated with caution because of the small sample size and the lack of detail on the statistical techniques used. The risk of overfitting needs to be considered. Although further research should be continued to improve technical issues, ethics should be taken into account. Martinez-Martin et al [115] questioned whether it is ethical to use prognostic estimates from ML to treat psychosis, as it is not known whether variables are present in the local context (such as differences in psychiatric practice and social support) that would affect the model’s validity. Moreover, when programming an ML algorithm, investigators can choose to strengthen the criteria they esteem to be more relevant, such as clinical criteria instead of socioeconomic factors. This could result in loss of opportunity for some patients when the automated machine analysis gives the illusion of greater objectivity. These adjustments should be done to respect the principle of equity.

In the case of predicting psychosis, the study involved only patients who consented to both psychiatric care and the completion of interviews. This was not the case in studies on suicide prevention, where researchers tracked information on patients by using social media. This could be considered a violation of confidentiality. Should information from social media be used to identify symptoms? Applying AI in this context raises significant ethical concerns, particularly in balancing beneficence and respecting confidentiality [53]. ML and NLP can help identify people at clinical risk for depression or suicidal ideation, who most likely do not have access to mental health providers and/or a primary care doctor [61]; however, this reduces confidentiality protection and can lead to increased vulnerability in certain populations [21]. To obtain informed consent from patients and protect their privacy, McKernan et al [53] proposed some recommendations: patients should be informed that (1) algorithms can be imperfect or wrong; (2) algorithm data should be considered highly sensitive or confidential; (3) algorithm data might recommend actions that are not immediately apparent; and (4) algorithms might prompt unnecessary intervention from the provider. Therefore, psychiatrists should be trained in ML and NLP techniques and be able to explain to patients their main characteristics and why they may require certain recommendations. This last point underlines the need for an explainable AI that goes further than black box methods.

Finally, ML and NLP should not lead to disempowerment of psychiatrists or replace the clinician-patient pair. On the contrary, the combination of ML with NLP should be considered as a *tool* to support clinical practice and medical research.

### Conclusions

In the past decade, the use of ML and NLP has become increasingly widespread in medicine and more specifically in psychiatry. Hence, this review aimed to summarize and characterize studies that used ML and NLP techniques for mental health in methodological and technical terms. The secondary aim was to consider the potential use of these methods in mental health clinical practice (eg, contribution to diagnosis, prognosis, establishment of risk factors, impact of psychotherapy, treatment adherence, and side effects).

Although the selected studies were heterogeneous in terms of topics and mental disorders, common features were found in terms of population categories (patients included in medical databases, patients presenting to the emergency room, and social media network users) and objectives (ie, symptom extraction, severity classification, comparison of therapies, findings of psychopathological clues, and challenges to the current nosography). The type-of-data-used analysis identified 2 major corpora: data collected by care providers (EHR, clinical notes, or EMR) and data from social media. Finally, the method analysis indicates that the authors privileged certain techniques. The standard methods of NLP (such as lemmatization, POS tagging, or n-grams) are most frequently used for preprocessing, in addition to CUI extraction dedicated to medical texts. The classification analysis specifies that classifiers with good performance (SVM, LogIR, and RF) are preferred to those with *transparent* functioning. The use of the universal programming language platforms such as Python and R is verified; Python turned out to be the most frequently and recently used. The correspondence analysis of data, methods, classifiers, platforms, and publications reveals a cluster of publications associating clinical notes data with cTAKES methods and the R-Python platform.

ML and NLP methods may sometimes be impressive with their huge amount of data screening and the multiple perspectives they offer. This has led some authors to consider it to be a new paradigm in mental health research. However, these processes tend to confirm clinical hypotheses rather than developing new information, and some results should be treated with caution (eg, results from social media users’ cohorts or the impact of language-specific features on NLP methods performance). On the contrary, ML and NLP techniques provide information from unexplored data and on patients’ daily habits that are usually inaccessible to care providers. It may be considered as an additional tool in every step of mental health care: diagnosis, prognosis, treatment efficacy, and monitoring. In this regard, ethical issues, such as predicting psychiatric troubles or implications in the physician-patient relationship, remain and should be discussed in a timely manner. Therefore, ML and NLP methods may offer multiple perspectives in mental health research, but they should be considered as a tool to support clinical practice.

## Appendix

Multimedia Appendix 1 Preferred reporting items for systematic reviews (PRISMA) flow diagram.

Multimedia Appendix 2 Table summarizing the selected studies.

## Abbreviations

AI: artificial intelligence
ASD: autism spectrum disorder
CEGS N-GRID: Centers of Excellence in Genomic Science Neuropsychiatric Genome-Scale and RDoC Individualized Domains
cTAKES: clinical Text Analysis and Knowledge Extraction System
CUI: concept unique identifier
DSM: Diagnostic and Statistical Manual of Mental Disorders
EHR: electronic health record
EMR: electronic medical record
LDA: latent Dirichlet allocation
LogiR: logistic regression
LSA: latent semantic analysis
ML: machine learning
NLP: natural language processing
NN: neural network
POS: part of speech
PTSD: posttraumatic stress disorder
RDoC: Research Domain Criteria
RF: random forest
SVM: support vector machine
UMLS: unified medical language system
