# Supplementary figures and images for: Machine Learning and Natural Language Processing in Mental Health: Systematic Review
Source: J Med Internet Res. 2021 May 4;23(5):e15708. doi: 10.2196/15708 (PMC8132982; doi:10.2196/15708)

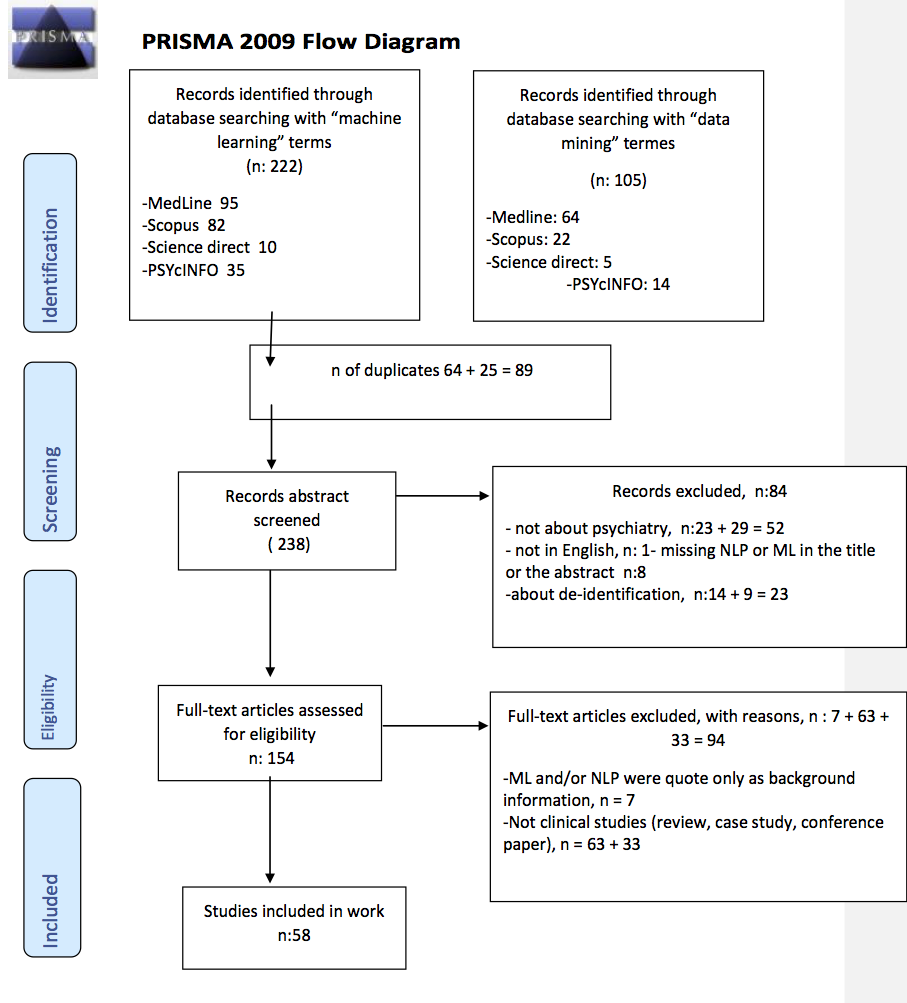

Supplement: Multimedia Appendix 1 [file jmir_v23i5e15708_app1.png]
