# Supplementary material for: Machine Learning and Natural Language Processing in Mental Health: Systematic Review
Source: J Med Internet Res. 2021 May 4;23(5):e15708. doi: 10.2196/15708 (PMC8132982; doi:10.2196/15708)
Supplement: Multimedia Appendix 2 [file jmir_v23i5e15708_app2.pdf]

A Table

Hart et al. 2020 [94]			
**Context**	**Population**	**Data**	**Volume**
As medication adherence remains a challenge in psychiatry, the study aims to mine sentiment vs. medication on the Web	Authors of Web pages containing specific generic and trade names of medications	Web pages	12,733 web-pages of minimum 100 words and maximum 30k words
**Goal:** To mine sentiment vs. 51 psychotropic medications across 3 drug classes (antidepressants, antipsychotics and mood stabilizers)			
**Method:** Web crowl on a curated list of medications (either generic or trade but not both together). Then sentiment analysis with VADER. Apparently the sentiment analysis is uniform on the document and does not take the medication name into account. No classification, simple statistical tests on medication+sentiment combinations (t-test, ANOVA). R platform used.			
**Results:** Sertraline, duloxetine, venlafaxine and paroxetine have a significantly greater negative sentiment than antidepressants, Quetiapine and risperidone have the most negative sentiment among antipsychotics, Lithium preparations and valproate exhibited less negative sentiment than other mood stabilizing medications.			

Glauser et al. 2019 [95]			
**Context**	**Population**	**Data**	**Volume**
Identification of suicidality or depression	122 individuals older than 12 years (average age 18 years), with epilepsy divided in three mutually exclusive groups: no psychiatric disorders, non-suicidal psychiatric disorders, any degree of suicidality	MINIs (Mini International Neuropsychiatric Interviews) or MINI Kid Trackings + 5 open-ended conversa- tional questions	122 first interviews and 105 second interviews
**Goal:** To create machine-learning classifiers that identify current or lifetime history of comorbid psychiatric conditions			
**Method:** LIWC and n-grams produced 21,603 features, classification with SVM. R platform used.			
**Results:** AROCs for models differentiating the non-overlapping groups and individual disorders ranged 57%-78% (many with p = 0.02).			

Guntuku et al. 2019 [96]			
**Context**	**Population**	**Data**	**Volume**
Loneliness as a major public health epidemic, depression and anxiety	Tweeter users	Tweets	400 million tweets
**Goal:** To establish a correlation between occurrences of ‘lonely’ or ‘alone’ in the users’ timelines and mental health			
**Method:** Four sets of language features: open vocabulary (LDA), dictionary-based (LIWC), mental well-being attributes (text regression), temporal patterns and use of drug words. Classification by random forests. Platform unknown.			
**Results:** Terms “lonely” and “alone” are associated with difficult interpersonal relationships, psychosomatic symptoms, substance use, desire of change, unhealthy eating and having troubles with sleep, anger, depression and anxiety			

To et al. 2019 [97]			
**Context**	**Population**	**Data**	**Volume**
Alcohol misuse in hospitalized patients	Non-trauma inpatient with alcohol use disorders (ICD diagnosis) and patients at-risk for alcohol misuse	Clinical notes in EHRs	1,000 patients
**Goal:** To identify cases of alcohol misuse in trauma patients			
**Method:** CUIs in UMLS are extracted by a logistic regression with LASSO (cTAKES tool). Classification also uses logistic regression with LASSO. Python platform.			
**Results:** The alcohol misuse classifier had an area under the receiver operating characteristic curve of 0.91 (95% CI 0.90-0.93) in the cohort of hospitalized patients			

Barroilhet et al. 2019 [98]			
**Context**	**Population**	**Data**	**Volume**
Personality disorder characterization in EHRs	Patients of an adult psychiatry inpatient unit at Massachusetts General Hospital between 2010 and 2016.	EHRs	4,702 admissions for 3,623 individuals
**Goal:** To characterize a psychiatric inpatient cohort in terms of personality trait in order to avoid inconsistent assessment			
**Method:** First choice of a list of “curated” terms based on expert consensus and then topic detection by LDA and choice of the topic with the highest probability for the curated terms. R platform used.			

**Results:** Male non-white individuals having a low burden of medical comorbidity, being admitted through the emergency department, and having public insurance, were independently associated with greater levels of disinhibition, detachment, and psychoticism. Female white individuals having private insurance were independently associated with greater levels of negative affectivity. The presence of disinhibition, psychoticism, and negative affectivity were each significantly associated with a longer stay, while detachment was associated with a shorter stay. The study has proven that personality features can be systematically and scalably measured using NLP in the inpatient setting, and some of these features can be associated with length of stay.

**Topaz et al. 2019 [99]**			
**Context**	**Population**	**Data**	**Volume**
Alcohol and substance abuse	Adult patients admitted to the intensive care units (ICUs) at the Beth Israel Deaconess Medical Center from 2001 to 2012	Clinical notes, including physician-written discharge summaries and nursing notes (admission notes, daily progress, status update notes, case management notes)	51,201 physician's notes, 412,343 nurse's notes
**Goal:** To extract alcohol and substance abuse status from physicians and nurses clinical notes			
**Method:** Corpus based on clinical notes. CUIs extraction, word and phrase embeddings, classification by random forests. R platform used.			
**Results:** in situ software NimbleMiner slightly outperformed other NLP systems (average F-score = .84) while requiring significantly less time for the algorithm's development. The study underlines the importance of nursing data for the analysis of electronic patient records.			

**Ambalavan et al. 2019 [100]**			
**Context**	**Population**	**Data**	**Volume**
Suicide	Suicide survivors expressing themselves on the Reddit social network	Online communications on the topic of suicide in the social networking platform, Reddit.	6,229 comments, 12,782 sentences
**Goal:** To detect suicide attempts and methods in Reddit comments			
**Method:** Lemmatization, 3-grams, POS-tags (also customized ones). Classification via SVMs, logistic regression, SGD and perceptron. Python NLTK platform used.			
**Results:** Classification method performance varies between suicide experiences, with F1-scores up to 0.92 for "drugs" and greater than 0.82 for "hanging" and "other methods". The exploratory analysis reveals that the most frequent reported suicide methods are drug overdose, hanging, and wrist-cutting.			

**Bittard et al. 2019 [101]**			
**Context**	**Population**	**Data**	**Volume**
Suicide	Patients registered in the Clinical Record Interactive Search (CRIS) system (South London and Maudsley). Cohort of suicide-related admissions	EHRs: unstructured text data, including total document counts for the 30 days prior to but excluding the day of admission	17,640 patients, 4,235 suicide-related admissions between 2006 and 2017 in the final dataset. Controls: 16,940
**Goal:** To elaborate an accuracy classifier in suicide risk assessment			
**Method:** SVM on 14 categorical features and 68 features manually extracted from text (patient having disturbed sleep, etc.) and tfidf on the entire EHRs. GATE platform used.			
**Results:** Best performance (precision 61%, recall 63%, $p < 0.001$) was obtained by combining all three types of features: categorical data from EHRs, features extracted from text and tfidf of entire EHRs.			

<table>
<tr><th colspan="4">Yan et al. 2019 [102]</th></tr>
<tr><th>Context</th><th>Population</th><th>Data</th><th>Volume</th></tr>
<tr><td>Eating disorders</td><td>Reddit users posting on specific subreddits</td><td>Posts of more than 10 words from six eating disorder-related subreddits</td><td>4,812 posts</td></tr>
<tr><td colspan="4">Goal: To detect posts reflecting a heightened risk of imminent plans to engage in disordered behaviors</td></tr>
<tr><td colspan="4">Method: Lowercasing, stop-words removal, numbers removal, stemming, n-grams, embedding. Logistic regression, PU learning and Word mover's distance used as classification methods. Based on Python NLTK platform.</td></tr>
<tr><td colspan="4">Results: Best results obtained by a classifier trained on 53 (38 positive and 15 negative) labeled posts (tfidf and logistic regression) and a classifier using the average of distances of words (in the embedding) to the closest five positive posts (among 38 positive posts).</td></tr>
</table>

<table>
<tr><th colspan="4">Danielsen et al. 2019 [103]</th></tr>
<tr><th>Context</th><th>Population</th><th>Data</th><th>Volume</th></tr>
<tr><td>Mechanical restraint</td><td>Patients mechanically restrained in the period between one hour and 3 days after the admission in psychiatry</td><td>Danish EHRs (MidtEPJ) unstructured clinical notes</td><td>5,050 patients with a total of 8,869 admissions</td></tr>
<tr><td colspan="4">Goal: To investigate whether incident mechanical restraint occurring in the first 3 days following admission can be predicted based on analysis of electronic health data available after the first hour of admission</td></tr>
<tr><td colspan="4">Method: Data are EHR with a few notes in natural language for which topic detection is applied but apparently the topics are not used for classification. POS, lemmatization, only nouns, verbs, adjectives and pronouns kept. Classification by logistic regression, LASSO, neural networks, Random Forest, SVM. SAS platform used.</td></tr>
<tr><td colspan="4">Results: Best performance by a Random Forest algorithm that predicted MR with an area under the curve of 0.87 (95% CI 0.79–0.93).</td></tr>
</table>

<table>
<tr><th colspan="4">Mezuk et al. 2019 [104]</th></tr>
<tr><th>Context</th><th>Population</th><th>Data</th><th>Volume</th></tr>
<tr><td>Suicide</td><td>Suicides and undetermined deaths among adults (55 years old and older) living in or transitioning to residential long-term care and listed in the National Violent Death Reporting System (NVDRS)</td><td>Narratives abstracted from coroner reports</td><td>47,759 deaths including 42 576 suicides, 279 deaths due to unintentional firearm injury, and 4904 undetermined deaths</td></tr>
<tr><td colspan="4">Goal: To estimate the number of suicides associated with residential long-term care among adults 55 and older. To identify whether machine learning tools could improve the quality of suicide surveillance data.</td></tr>
<tr><td colspan="4">Method: Search of terms (curated list) and then training on the corpus of documents containing the terms, with tfidf weights. Then classification via random forests. Python NLTK platform used.</td></tr>
<tr><td colspan="4">Results: Among 47,759 deaths, the algorithm identified 1,037 associated with long-term care</td></tr>
</table>

<table>
<tr><th colspan="4">Carson et al. 2019 [105]</th></tr>
<tr><th>Context</th><th>Population</th><th>Data</th><th>Volume</th></tr>
</table>

Suicide	Adolescents hospitalized on a psychiatric inpatient unit in a community health system in the northeastern United States	Unstructured clinical notes were downloaded from the year preceding the index inpatient admission	73 respondents
**Goal:** To identify suicidal behavior among psychiatrically hospitalized adolescents			
**Method:** Detection of CUI UMLS terms (and negation) in texts (Invenio system, based on cTAKES), named entity recognition, then use of random forest as classifier. Using R platform.			
**Results:** An AUC of 0.68 for prediction of suicide attempts in EHRs			

**Parthipan et al. 2019 [106]**			
**Context**	**Population**	**Data**	**Volume**
Depression and postoperative pain	Surgical patients with symptoms of depression	EHR data (e.g., medications, vitals, demographics) captured before surgery	4,306 patients
**Goal:** To predict inadequate postoperative pain in depressed patients			
**Method:** Removal of stop-words and words of less than 2 letters, conversion of numbers into strings, CUIs UMLS extraction (in-. Classification via logistic regression (ElasticNet), ridge regression and LASSO. Python NLTK platform used.			
**Results:** Prediction of increase or decrease of post-operative pain at discharge, 3-week and 8-week follow-up with AUC 0.87, 0.81 and 0.69 respectively			

**Afshar et al. 2019 [81]**			
**Context**	**Population**	**Data**	**Volume**
Alcohol misuse	Patients admitted to a trauma center between April 2013 and November 2016	Unstructured clinical notes and 16 features (age, sex, ethnicity, alcohol dependence, mechanism of injury, etc.)	91,045 electronic health record notes (22,642 available in the 24 hours from patients admission) concerning 1,422 adult patients
**Goal:** To identify alcohol misuse from the electronic health record in trauma patients			
**Method:** Questionnaire by the WHO on alcohol consumption and tfidf on clinical notes to detect CUIs UMLS (and their negations) using cTAKES. For classification, many algorithms tested incl. logistic regression and SVM. Python scikit-learn platform used.			
**Results:** Best AUC result by logistic regression: 0.78 with the following top positive CUIs: thia- mine, intoxication, neglect, drinking problems, drinking, liver imag- ing, sexually active, marijuana, and alcohol or drug abuse			

**Vaucheret et al. 2019 [107]**			
**Context**	**Population**	**Data**	**Volume**

Neurodevelopmental disorders	Subjects between 9 and 17 years of age with neurodevelopmental disorders divided in 3 groups: attention deficit hyperactivity disorder (ADHD), autism spectrum disorder (ASD), learning disabilities (LD), and intellectual disability (ID) groups	Neuropsychological assessment: Intelligence test (Wechsler Scale, version WISC V in Spanish), verbal fluency tests of the neuropsychological battery NEPSY II, verbal fluency subtest with semantic and phonological tasks, and Sally-Anne Theory of Mind test by Baron-Cohen. Interviews after viewing 2 soccer videos: thoughts and feelings about them	37 subjects
**Goal:** To analyze sentiment in children with neurodevelopmental disorders			
**Method:** Google Sentiment Analysis tool was applied to small Spanish texts written by the subjects after watching videos. Chi2, Fisher test, linear regression. Stata software used.			
**Results:** Although everybody knew the rules of soccer, when the participants punished the transgressor, a preference for members of their own group was observed, except for the ASD group. Children with ASD seem not to base their opinion on their group membership, but rather on precise adherence to regulations.			

**Zhong et al. 2019 [85]**			
**Context**	**Population**	**Data**	**Volume**
Suicide of pregnant women	Partners HealthCare System Research Patient Data Registry, a clinical data warehouse that gathers medical records for nearly 4.6 million patients from Massachusetts General Hospital (MGH) and Brigham and Women's Hospital (BWH)	Ambulatory notes, discharge summaries, EPIC progress reports (such as emergency department (ED) observation progress notes, labor and delivery notes, lactation notes, progress notes, and significant event notes), operative notes. Pathology, cardiology, endoscopy, pulmonary, and radiology reports.	273,410 women with at least one CUI related to pregnancy or delivery, of which 23,098 with mention of CUIs related to suicidal behavior during pregnancy or within the 42 days after abortion or delivery
**Goal:** To develop a classification algorithm that would accurately identify pregnant women with suicidal behavior			
**Method:** CUIs UMLS extraction via cTAKES and additional suicide-related features added manually. Classification using logistic regression (elastic net). R platform used.			
**Results:** Best AUC value: 0.83 for an algorithm using ICDs, extracted CUIs and additional expert-curated features: feeling hopeless, feeling relief, tired, love, feeling empty, feeling content, low self-esteem, impulsive character, isolation, distractibility, childhood adversity, adult sexual abuse, severe depression, substance abuse problem, personality disorders, psychotic disorders, seizures, anxiety disorders, wound and injury, abortion.			

**Leroy et al. 2018 [58]**			
**Context**	**Population**	**Data**	**Volume**
Autism	4 to 8-year old children from 11 US states	EHRs, behavior descriptions by clinicians.	1st test: 50 EHRs (6,636 sentences), 2nd test: 4,480 EHRs
**Goal:** To automatically extract DSM criteria for ASDs from EHRs			

**Method:** CUIs UMLS extraction. Bag-of-words, lemmas appearing at least 5 times in the corpus. Classifier: pruned decision tree on the sentences (Weka platform). Pattern extraction using JAPE (finite-state transducers) in the frame of the GATE platform.
**Results:** Annotation: 74% precision and 42% recall, sentence-level evaluation: 76% precision and 43% recall. Furthermore, the parser achieved 82% precision and 46% recall in identifying 1,357 sentences annotated for autism-like behavior

**Coppersmith et al. 2018 [84]**			
**Context**	**Population**	**Data**	**Volume**
Suicide	OurDataHelps.org users endorsing suicide attempts, and users publicly discussing past suicide attempts on social media (predominantly females ages 18-24).	Posts from multiple social media platforms (Facebook, Twitter, Instagram, Reddit Tumblr)	418 individuals for which there are at least six months of posts prior to suicide attempt available (in average 473 posts per individual).
**Goal:** To create an automated model for analysis and estimation of suicide risk from social media data			
**Method:** Sequences of word vectors using gloVe embedding. Clasifier: BiLSTM network with attention mechanism. Platform unknown.			
**Results:** AUC of 0.94			

**Duy Van Le et al. 2018 [108]**			
**Context**	**Population**	**Data**	**Volume**
Violence in an inpatient forensic psychiatry setting	Entries recorded in the Wilfred Lopes Center (WLC) mental health service (Tasmania)	EHRs for inpatients sicne October 2006 + HCR-20 (Historical Clinical Risk Management-20), START (Short-Term Assessment of Risk and Treatability) and DASA (Dynamic Appraisal of Situational Aggression) scores	220,000 entries (20M words), 193 HCR-20, 300 START and over 18,000 DASA.
**Goal:** To determine which dictionaries in conjunction with which ML algorithms allow assessment of risk of violence self or others			
**Method:** Four dictionaries (symptoms, diagnosis, sentiment, frequency) have been created/selected manually. CUIs UMLS extraction (MedLEE, cTAKES). Classification by logistic regression, SVM, bagging, J48, Jrip and LMT.			
**Results:** The most accurate prediction (0.61) was attained using the sentiment dictionary and SVM algorithm. CLAMP platform used [93]			

**Chary et al. 2018 [67]**			
**Context**	**Population**	**Data**	**Volume**
Addiction to LSD+Ecstasy	Lycaeum (social network) users	Posts on Lycaeum from its start in 1996 to 2016	9,289 posts
**Goal:** To identify drug-drug combinations from online users' posts			
**Method:** Lemmatization, stop-words removal. Manual detection of drug names from a list of nouns. Calculation of co-occurrences of these terms in the documents. Python NLTK platform used.			
**Results:** 183 drug-drug combinations detected, out of which 44 have never been directly studied but are similar to combinations that have been directly studied and three (nefazodone & pramipexole, zacatechichi (mugwort) & skullcap, and niacin & GABA) have no antecedents in the literature.			

Fernandes et al. 2018 [60]			
**Context**	**Population**	**Data**	**Volume**
Suicide	Patients registered in the Clinical Record Interactive Search (CRIS) system (South London and Maudsley). Suicide attempt cohort, suicidal ideation cohort.	EHRs: free-text and correspondences between patient and clinical staff	500 documents selected out of 188,843 from a suicidal ideation cohort, and 500 documents selected out of 542,769 from a suicide attempt cohort.
**Goal:** To develop two NLP tools (one for detecting the presence of recorded suicidal ideation, and one for detecting a recorded suicide attempt) and to compare them with manual text annotation.			
**Method:** EHRs and correspondence, bag-of-words, POS tags, stemming, detection of negation, mention of another person, temporal irrelevance. Manually constructed list of 150 terms. Classification by SVM. GATE platform used.			
**Results:** The rule-based algorithm achieved a sensitivity of 87.8% and a precision of 91.7%. The hybrid algorithm achieved a sensitivity of 98.2% and a precision of 82.8%.			

Yazdavar et al. 2018 [86]			
**Context**	**Population**	**Data**	**Volume**
Depression	Twitter profiles with at least 100 tweets emitted	Tweets	4,000 Twitter users randomly selected (the half of which taken from a set of 7,046 users (21M tweets) having self-declared their depression, and the other half having no depression terms in their profile descriptions)
**Goal:** To detect depressive symptoms on Twitter profil			
**Method:** Use of lexicon and of topics. LDA being insufficient, supervision is added to it (FOL LDA by Andrzejewski 2011), so that terms "strongly related" to a list of curated terms are detected. The algorithm (ssToT) is described in the paper. It is applied to tweets, and topic coherence is measured. The method is compared with other topic detection methods. Python NLTK platform used.			
**Results:** ssToT model allowed identification of clinical depressive symptoms with an accuracy of 68% and a precision of 72%. The ssToT model is competitive with supervised approaches in terms of F-score.			

Maguen et al. 2018 [63]			
**Context**	**Population**	**Data**	**Volume**
Evidence-based psychotherapy in PTSD	255,933 veterans of Iraq or Afghanistan wars with PTSD diagnosis	Clinical notes from psychotherapy visits	8,168,330 clinical notes
**Goal:** To detect and discriminate between note texts describing evidence-based protocols for PTSD and other psychotherapies.			
**Method:** Bag-of-words, removal of irrelevant terms, manually created set of salient terms. Classifier: linear SVM trained on manually annotated notes. Apache UIMA platform used.			
**Results:** Acceptable level of performance with PE accuracy of 0.99, CPT individual and CPT group accuracy of 0.97 and overall classification accuracy of 0.92. 20% of Iraq and Afghanistan veterans received at least one session of EBP for PTSD over 15 years of observation.			

Corcoran et al. 2018 [109]			
**Context**	**Population**	**Data**	**Volume**

Psychosis	Participants meeting criteria for one of three prodromal syndrome categories of SIPS/SOPS.	Transcribed speech (Caplan's *Story game*), open-ended narrative interviews.	93 participants (59 from Los Angeles and 34 from New York)
**Goal:** To predict psychosis.			
**Method:** Speech transcripts were lemmatized, punctuation removed, POS-tagged and then converted to vectors through LSA. Frequencies of comparative adjectives, possessive pronouns and WH-words were calculated. Classifiers: singular value decomposition and logistic regression. Python NLTK platform used.			
**Results:** Accuracy of the classifier trained on Los Angeles data on Los Angeles data: 83%, accuracy on the New York dataset: 79%.			

**Roy et al. 2018 [40]**			
**Context**	**Population**	**Data**	**Volume**
Cyber-based harassment (digital dating abuse)	Anonymous teenagers and young adults having written stories on MTV's Web site. 44 participants from Clemson University (mean age 20.5 years, 87% male) to author stories based on them. Victims of abusive relationships, family members of victims and researchers annotated them.	Abusive and non-abusive text messages	Selection of 70 initial stories out of 728 MTV stories. 161 abusive text messages authored our of them. 140 non-abusive messages extracted from the SMS Spam Corpus and/or the Mobile Forensics Text Message Corpus.
**Goal:** To create an initial training set for digital dating abuse, and to classify text messages as abusive or non abusive.			
**Method:** Lemmatized, count-vectorized, tfidf. Classification by SVMs (linear), multinomial NB and decision trees, on *n*-grams (for $n \leq 3$). Python scikit-learn platform used.			
**Results:** Best accuracy (0.89) achieved by linear SVM, unigrams and tfidf weights.			

**Moessner et al. 2018 [110]**			
**Context**	**Population**	**Data**	**Volume**
Eating disorders	Reddit users	Reddit posts	An ad-hoc dataset of 4,247 posts and 34,118 comments by 3,029 users of the proed forum on Reddit.
**Goal:** To analyze data on an eating-disorder social forum.			
**Method:** Punctuation and numbers, stop-words and hapaxes removed, then LDA applied (with 9 and 11 topics). No classification, just Speerman correlation between topics. R platform used.			
**Results:** The aim was not to report results but to demonstrate strategies and the potential of big data approaches in social media.			

**Doan et al. 2017 [41]**			
**Context**	**Population**	**Data**	**Volume**
Stress/Anxiety	Twitter users in Los Angeles, New York, San Diego, San Francisco	Tweets	2,107 tweets (1,326 containing #stress and 781 containing #relax)

**Goal:** To understand how people express their feelings of stress and relaxation through Twitter messages. To classify stress or nonstress and relaxation or nonrelaxation tweets. To identify first-hand experience in stress and relaxation tweets.
**Method:** Extraction of tweets containing the hash tags #stress and #relax, then bag-of-words, unigrams/bigrams, and relevance/theme classification with Naïve Bayes and SVMs. Bow platform (Carnegie Mellon University) used.
**Results:** Twitter users posted more about the cause of their stress and less about symptoms associated with stress. Education was the most frequent topic of stress, vacation was the most frequent topic of relaxation. SVM performed better in classifying stress vs non stress tweets (81.66% accuracy), idem for relaxation (83.72% accuracy) but NB performed better for first-hand experience detection (87.58% accuracy).

**Tran et al. 2017 [72]**			
**Context**	**Population**	**Data**	**Volume**
Transnosographic CEGS N-GRID 2016 (3rd track: Novel Data Use)	Patients randomly selected from Partners Health Care	Neuropsychiatric clinical records	986 records
**Goal:** To predict the binary presence (yes/no) of mental conditions based on the history of present illness: ADHD, Anxiety, Bipolar, Dementia, Depression, Eating disorder, Grief, OCD/OCSD, Panic, Psychosis, PTSD.			
**Method:** Corpus based on medical notes for which the history of present illness is known. 11 labels have been manually selected. They are used for predictions with various models; NER of psychiatric conditions, CNN, RNN with hierarchical attention (ReHAN), BiLSTM, as well as SVMs. Python Theano platform used.			
**Results:** Depending on the target label results are quite different: for anxiety, NER achieves best precision (75.7%) and CNN best recall (96.4%); for depression, NER achieves best precision (84.7%) and ReHAN best recall (99.3%); for psychosis, CNN achieves best precision (69.3%) and SVM best recall (ony 25.5%), etc.			

**Scheurwegs et al. 2017 [73]**			
**Context**	**Population**	**Data**	**Volume**
Transnosographic CEGS N-GRID 2016 (2nd track: RDoC classification)	Patients randomly selected from Partners Health Care	Neuropsychiatric clinical records	816 records
**Goal:** To create a new method for identifying the severity associated with a patient's positive valence symptoms from a set of psychological evaluation records.			
**Method:** CUIs UMLS extraction (cTAKES). Negation and irrelevant information are removed by manually written rules. A bag-of-words approach is applied in parallel to a UMLS-based approach. UMLS were embedded by taking the mean of individual words contained in them. Bootstrapping and outlier removal is applied to the data set. Classification uses random forests. Platform Python sci-kit learn used (code available on github).			
**Results:** Best result (MAE 80.64%) obtained by use of subset of UMLS based on DSM-IV ontology, with context, bootstrapping and outlier removal. The "absent" and "mild" severity labels are predicted best.			

**Goodwin et al. 2017 [59]**			
**Context**	**Population**	**Data**	**Volume**
Transnosographic CEGS N-GRID 2016 (2nd track: RDoC classification)	Patients randomly selected from Partners Health Care	Neuropsychiatric clinical records	816 records
**Goal:** To create a new method for identifying the severity associated with a patient's positive valence symptoms from a set of psychological evaluation records.			

**Method:** Sentence splitting, lemmatization, negation span detection, CUIs in UMLS and ICD-9. Selection of relevant parts in EHRs (questions answered by yes, non-negated narrative content, elaboration of questions answered by no). Classification by three methods: ridge regression, pairwise random forest, aggregation of these two. These methods provide a continuous score, use of cascading SVM trees in order to map it into four classes. Platform Python scikit-learn used.
**Results:** Hybrid model (point-wise ridge regression and pairwise random forest classification) performed best (MAE 84.10%). The severity labels on the extremes "absent" and "severe" were the easiest to classify while distinguishing between "mild" and "moderate" was more difficult.

**Posada et al. 2017 [74]**			
**Context**	**Population**	**Data**	**Volume**
Transnosographic CEGS N-GRID 2016 (2nd track: RDoC classification)	Patients randomly selected from Partners Health Care	Neuropsychiatric clinical records	816 records
**Goal:** To develop a framework to automatically classify initial psychiatric evaluation records from one to four positive valence system severity: absent, mild, moderate, or severe.			
**Method:** CUIs UMLS extraction (MedLEE). Stop-word and punctuation removed, words lemmatized. Questions relevant to the task were manually selected and their answers modeled. The set of all features obtained was classified by two decision-tree models, a Bayesian network, a hierarchy-based Bayesian network and SVMs applied to tfidf weights. Platform R used.			
**Results:** Best predictive scores was achieved by decision trees (MAE 82.56%).			

**Takano et al. 2017 [111]**			
**Context**	**Population**	**Data**	**Volume**
Memory dysfunctions	Participants were recruited in a Japanese community by an online survey company.	Japanese version of AMT (Autobiographical Memory Test)	Study 1: 12,400 memories. Study 2: 8,478 memories. Study 3: data from both studies
**Goal:** To reveal the linguistic features of specific memories on the AMT. To create a computerized classifier that distinguishes between specific and non specific memories.			
**Method:** Corpus of small Japanese texts (autobiographical memories). Segmentation into morphemes, lemmatization, POS tagging, tfidf. Classification by Gaussian SVMs. Feature selection through χ^2. Platform R used.			
**Results:** Memory responses tend to be written in longer sentences and contain a richer vocabulary, more past-tense auxiliary verbs and past-tense expressions, than non-memory responses that contain less function words. Analysis is largely dependent on the Japanese language structure. Best result (AUC 0.92) is achieved by SVM classifier.			

**Jackson et al. 2017 [79]**			
**Context**	**Population**	**Data**	**Volume**
Transnosographic	South London and Maudsley NHS Trust (SLAM) patients	EHRs	More than 20M free-text documents for 230k patients
**Goal:** To create a free-text analysis tool able of extraction/training/analysis by ML algorithm. To identify a patient's cannabis smoking status. To identify the presence of delusional symptoms and evidence of hallucinations. To classify instances of text describing a patient's ethnicity into one of 17 ethnic groups.			
**Method:** Bag-of-words, POS, stemmed, with manually written rules for negation, temporal irrelevance, and reference to a person other than the patient. Classification by SVM. Platform GATE used.			
**Results:** The best performance was seen in the hallucinations case study with 97% recall obtained at the 95% precision threshold. Worst performance with ethnicity study with 9% recall at the 90% precision threshold.			

**Filannino et al. 2017 [76]**			
**Context**	**Population**	**Data**	**Volume**
Transnosographic CEGS N-GRID 2016 (2nd track: RDoC classification)	Patients randomly selected from Partners Health Care	Neuropsychiatric clinical records	816 records
**Goal:** To determine the lifetime maximum symptoms of severity of patient's mental disorders, based on the information reported in their initial psychiatric evaluation.			
**Method:** Description of a shared task on neuropsychiatric EHRs. The study reviewed 65 submissions from 24 teams which submitted different classification algorithms.			
**Results:** Best models were Metrix Inc (ensemble of SVM machine with RBF kernel, Random Forest, Multinomial Naive Bayes, Adaboost, Deep Neural Network) compared with gold standard (manual classification). MAE of 0.86.			

**Clark et al. 2017 [75]**			
**Context**	**Population**	**Data**	**Volume**
Transnosographic CEGS N-GRID 2016 (2nd track: RDoC classification)	Patients randomly selected from Partners Health Care	Neuropsychiatric clinical records	816 records
**Goal:** To elaborate a new method for identifying the severity associated with a patient's positive valence symptoms from a set of psychological evaluation records.			
**Method:** Bag-of-word approach (no stemming or lemmatization), manual creation of a list of terms, creation of word2vec embeddings and K-Means applied to them. Other features: DSM codes and Yes/No answers to specific questions. In total, 29,375 features. Classifier: 5-layer neural network. Apache Spark Mandolin platform used.			
**Results:** Best result achieved by multi-layered perceptron (MAE 77.86%).			

**Coppersmith et al. 2017**			
**Context**	**Population**	**Data**	**Volume**
Transnosographic	Employees of a company	Internal chat, communications and file sharing	Not mentioned
**Goal:** To analyse whitespace information (data available between interactions with health care system) to provide psychological phenomena like emotional crises, suicide attempts, and drug relapse.			
**Method:** Corpus of intracompany communications, chat and file sharing. Use of LIWC. Emotion analysis by *n*-grams (on the character level) and logistic regression. Sentiment analysis using LIWC, Naive Bayes and SVMs. Python sci-kit learn platform used.			
**Results:** The company exhibits increases in "joy" around the major holidays and after the first major software release on a new project, and exhibit increases in negative sentiment leading up to each major deadline.			

**Hoogendoorm et al. 2017 [112]**			
**Context**	**Population**	**Data**	**Volume**
Social anxiety	Patients diagnosed with a social anxiety disorder	Socio-demographic data, free text (email), basic mailing behavior, word usage, writing style, sentiment,	69 patients
**Goal:** To predict therapeutic outcomes using writing from patients treated for social anxiety disorders			
**Method:** German language corpus. Analyzing mail discourse characteristics, tfidf on stemmed words (stop-words removed), writing style depending on POS tag patterns (no description of the patterns used), word-based sentiment analysis, topic modeling with LDA. Classification by CART, logistic regression and random forests. Python NLTK platform used.			

**Results:** Prediction outcome therapy with AUC of 0.83 halfway through the therapy and a precision of 0.78 during the entire treatment period.

**Dai et al. 2017 [87]**			
**Context**	**Population**	**Data**	**Volume**
Violence	Patients randomly selected from Partners Health Care	Neuropsychiatric clinical records (Transnosographic CEGS N-GRID 2016)	1,000 records
**Goal:** To establish associations of clinical and social parameters with violent behavior among psychiatric patients			
**Method:** Text spell-corrected. CUIs UMLS extraction (cTAKES). To detect violent behavior, 49 questions manually selected and extracted by manual rules. Then words "violent" and "violence" detected (considering also negation). Clinical data combined with linguistic data using association rule mining. Platform .Net used.			
**Results:** Stimulants, family history of violent behavior, suicidal behaviors, and financial stress are strongly associated with violent behavior.			

**Chary et al. 2017 [78]**			
**Context**	**Population**	**Data**	**Volume**
Misuse of Prescription Opioids	Tweeter users	Tweets	3,611,528 tweets
**Goal:** To demonstrate that the geographic variation of social media posts mentioning prescription opioid misuse strongly correlates with government estimates of MUPO (Misuse of Prescription Opioids) in the last month			
**Method:** Twitter corpus with geolocalization (Carmen). Lemmatization and removal of stop words, then WordNet-induced semantic similarity between tweets, K-Means with silhouette and PCA. Python NLTK platform used.			
**Results:** Mentions of MUPO on Twitter correlate strongly with state-by-state NSDUH estimates of MUPO. Natural language processing can be used to analyze social media to provide insights for syndromic toxicosurveillance.			

**Cook et al. 2016 [61]**			
**Context**	**Population**	**Data**	**Volume**
Suicide	Male or female, aged 18 or older discharged after self-harm from emergency services or after being hospitalized for less than 7 days, able to be contacted by phone.	Mobile application questionnaire (suicidal ideation, GHQ-12), reported information (hours of sleep, sleep quality, appetite, treatment adherence), WHO-5 questionnaire	1,453 patients
**Goal:** To predict patient's probabilities of suicidal ideation or heightened psychiatric symptoms (GHQ-12 ≥ 4)			
**Method:** CUIs UMLS extraction (cTAKES), *n*-grams and linear regression. Invenio platform used.			
**Results:** Structured data-based models perform better than NLP-based models for suicidal ideation and heightened psychiatric symptoms.			

**Tanana et al. 2016 [43]**			
**Context**	**Population**	**Data**	**Volume**
Motivational interviewing	Patients following motivational interviewing	Notes taken by clinicians in motivational interviewing sessions	1,7 million words.

**Goal:** To compare two NLP methods for MI automated coding
**Method:** The texts of psychotherapy sessions were manually labeled as a golden corpus. To automate the task, two approaches: (a) *n*-grams ($n \leq 3$) and dependency parse trees (Stanford parser), multinomial regression and (b) glove embedding using POS-dependent weights, RNNs. The platform emulab was used.
**Results:** Dependency trees performed equally well or better than RNNs.

**Luo et al. 2016 [48]**			
**Context**	**Population**	**Data**	**Volume**
Autism	27 adults with ASD and 132 matched controls	Written questionnaires with verbal descriptions of the patients' relatives	159 participants
**Goal:** To reveal patterns in descriptions of social relations by adults with ASD			
**Method:** What the authors call "semantic network" is in fact a graph of co-occurrences of words having the largest contributions to LSA dimensions. Density of the graphs is measured (called "connectivity" by the authors) as well as clustering coefficients. Classification with linear/quadratic regression and SVMs. Matlab platform used.			
**Results:** There is a difference in word connectivity patterns between the ASD and the typical participants, with the ASD participants' semantic network exhibiting less "small-world" characteristics.			

**Pestian et al. 2016 [62]**			
**Context**	**Population**	**Data**	**Volume**
Suicide	Patients admitted to ED with suicidal ideation, gestures, or attempts and their matched controls	Columbia Suicide Severity Rating Scale, Suicidal Ideation Questionnaire-Juniors, ubiquitous questionnaires, interviews	60 participants
**Goal:** To identify suicidal subjects in EDs.			
**Method:** Corpus of transcription of interviews. No information is given on text preprocessing (other than "converting it to a matrix" by which is meant a matrix with interviewees as rows and responses as cells). For classification, SVM has been chosen and then k-Means to show the shape of the two groups. Leave-one-out was used for testing/training corpus separation when the sets were small. Platform not mentioned.			
**Results:** The number of unique words was significantly different between suicidal and non-suicidal subjects. SVM classified 96.67% of the subjects accurately compared with the Columbia Suicide Severity Rating Scale (C-SSRS).			

**Metzger et al. 2016 [57]**			
**Context**	**Population**	**Data**	**Volume**
Suicide	Patients admitted to ED at Hôpital de la Croix-Rousse (Lyon) in 2011 and 2012, for suicide attempt or suicidal ideation plus a control group	French EHRs	966 records
**Goal:** To predict the annual rate of emergency department visits for suicide (compared to the national surveillance system based on manual coding by emergency pratitioners)			
**Method:** CUIs UMLS extraction. Seven classification methods (predictive association rules, decision trees, neural networks, logistic regression, random forests, SVMs, Naïve Bayes). R platform used.			

**Results:** Methods with the best F-measures were the random forest method (95.3%) and Naïve Bayes classifier (95.3%). The number of cases of suicidal ideation (false positive suicide attempts) detected by the random forest method was higher (94 vs. 93). Random forests, NB, SVM, association rules, decision trees estimated close to the gold standard method (manual classification) and would be valuable for epidemiological surveillance of suicide attempts.

**Tung et al. 2016 [113]**			
**Context**	**Population**	**Data**	**Volume**
Depression	Bloggers	Web posts from Chinese bulletin board PTT, between March 2004 and September 2011	18,000 posts collected out of which 724 were selected
**Goal:** To analyze and predict the depression tendency of Web posts.			
**Method:** Four dictionaries (negative event, negative emotion, symptom, negative thought), extraction of frequent POS patterns. Classification by log-linear regression. No platform mentioned.			
**Results:** Best method achieved a precision of 57.2% and a recall of 46.8% for negative event extraction, For depression tendency analysis, the model has better recall than legacy method (66.8% vs. 57.1%) but weaker precision (59.3% vs. 66.6%).			

**He et al. 2015 [47]**			
**Context**	**Population**	**Data**	**Volume**
PTSD	Trauma survivors	Free-text, demographic questionnaire.	300 participants
**Goal:** To overview the procedure of automated textual assessment on patients' self-narratives for PTSD screening. To compare the performances of different classification models in conjunction with *n*-gram representations in the screening process.			
**Method:** Bag-of-words, *n*-grams, stop-word removal, stemming, feature selection by χ^2, classification by decision trees, Naïve Bayes, SVM, product score model. Platform not mentioned.			
**Results:** Best recall (95%) obtained by trigrams and SVM. Best specificity (81%) by unigrams+bigrams and product score model.			

**Bedi et al. 2015 [82]**			
**Context**	**Population**	**Data**	**Volume**
Psychosis	Youth at clinical high risk for psychosis ascertained by SIPS/SOPS.	Open-ended narrative interviews of 1 hour	34 participants
**Goal:** To predict later psychosis onset in youth at clinical high-risk for psychosis.			
**Method:** Speech transcripts w/o punctuation, lemmatized, lower cased. Then phrase vectors embedded in 400-dimensional space through LSA. Coherence of consecutive phrases or of phrases with an intervening phrase measured. POS tags added to words. Convex hulls used for classification. Python NLTK platform used.			
**Results:** The convex hull classifier yielded 100% precision and recall for prediction of psychosis onset.			

**Roysden et al. 2015 [56]**			
**Context**	**Population**	**Data**	**Volume**
Health care utilisation after behavioral health referral	Patients who were seen at least once in a Brigham and Women's Hospital primary care pratice	EMRs	12,759 patients
**Goal:** To predict two outcomes following a patient's first behavioral health encounter: decreased utilization of healthcare, or ultra-high absolute utilization			

**Method:** *n*-grams ($n \leq 3$), removal of stop-words except for negating terms. Classification by random forests. Python scikit-learn used.
**Results:** Decreased utilization prediction: AUC 0.74. Ultra-high absolute utilization prediction: AUC 0.88

**Baggott et al. 2015 [66]**			
**Context**	**Population**	**Data**	**Volume**
MDMA	Healthy volunteers with prior MDMA experience	Transcribed speech: five-minute standardized talking tasks	35 participants
**Goal:** To investigate the effect of MDMA on speech content and reveal how this drug affects social interactions.			
**Method:** LIWC, bag-of-words, named entities removed, stemming. Classification by random forest. Python NLTK platform used.			
**Results:** MDMA alters speech content relative to placebo, it increases the use of social and sexual words, to both positive and negative emotions.			

**Carrell 2015 [80]**			
**Context**	**Population**	**Data**	**Volume**
Opioid use	Patients receiving chronic opioid therapy (≥ 70 days' supply of opioids per calendar quarter) during 2006-2012	EHRs	22,142 patients
**Goal:** To identify evidence of problem opioid use in EHRs			
**Method:** First construction of a dictionary of 1,288 terms related to opioids. Then CUIs UMLS extraction (cTAKES) while avoiding historical mentions, hypothetical mentions, references to others than the patient and negation. Python platform used.			
**Results:** Traditional diagnostic codes for problem opioid use were found for 10.1% patients. NLP assisted manual review identified an additional 3.1% patients with evidence of clinically diagnosed problem opioid use in clinical notes. Inter-rater reliability among pairs of abstractors reviewing notes was high, with kappa = 0.86 and 97% agreement for one pair, and kappa = 0.71 and 88% agreement for another pair.			

**Patel, Jayatilleke et al. 2015 [114]**			
**Context**	**Population**	**Data**	**Volume**
Negative symptoms in schizophrenia	South London and Maudsley NHS Trust patients with schizophrenia diagnosis receiving care during 2011	EHRs	7,678 patients
**Goal:** To identify negative symptoms in the EHRs of patients with schizophrenia and assess their relationship with clinical outcomes			
**Method:** Bag-of-words with special care for negation (positive or negative instances of negative symptoms). Classification by SVM and rules (hybrid). Stata software used.			
**Results:** 10 different negative symptoms were ascertained with precision above 0.80. 41% of patients had 2 or more negative symptoms. Negative symptoms were associated with younger age, male gender and single marital status, and with increased likelihood of hospital admission (OR 1.24, 95% CI 1.10 to 1.39), longer duration of admission (β-coefficient 20.5 days, 7.6-33.5), and increased likelihood of readmission following discharge (OR 1.58, 1.28 to 1.95).			

<table>
<tr><th colspan="4">Iqbal et al. 2015 [115]</th></tr>
<tr><th>Context</th><th>Population</th><th>Data</th><th>Volume</th></tr>
<tr><td>Adverse drug events</td><td>South London and Maudsley NHS Trust (SLaM) patients between 2007 and 2012</td><td>EHRs</td><td>200,000 patient records</td></tr>
<tr><td colspan="4">Goal: To identify instances of Adverse Drug Events (ADEs) from free-text psychiatric EHRs</td></tr>
<tr><td colspan="4">Method: Rules written manually to detect terms hinting to adverse drug effects, negation is taken into consideration. GATE platform used.</td></tr>
<tr><td colspan="4">Results: EPSEs with 0.85 precision and 0.86 recall during testing. Akathisia was found to be the most prevalent EPSE overall and occurred in the Asian ethnic group with a frequency of 8.13%.</td></tr>
</table>

<table>
<tr><th colspan="4">Patel, Lloyd et al. 2015 [116]</th></tr>
<tr><th>Context</th><th>Population</th><th>Data</th><th>Volume</th></tr>
<tr><td>Mood instability</td><td>South London and Maudsley NHS Trust (SLaM) patients between 2006 and 2013, aged between 16 and 65 years with a diagnosis of schizophrenia and related disorders, bipolar affective disorder, psychotic depression, personality disorder, unipolar depression without psychosis or any other affective disorder</td><td>Demographic information and unstructured free-text fields from case notes and correspondence</td><td>27,704 adults</td></tr>
<tr><td colspan="4">Goal: To assess the impact of mood instability on clinical outcomes in a large sample of people receiving secondary mental healthcare</td></tr>
<tr><td colspan="4">Method: Search for the left- and right-modifiers of words “mood,” “affect,” and “emotion”. Bag-of-words. An SVM was used to find relevant sentences. Then multiple linear regression was applied to predict the number of days spent in psychiatric hospital. Stata software used.</td></tr>
<tr><td colspan="4">Results: Mood instability was documented in 12.1% of people presenting to mental healthcare services (22.6% in people with bipolar disorder, 17.8% personality disorder, 15.5% schizophrenia). It was associated with a greater number of days spent in hospital (β coefficient 18.5, 95% CI 12.1 to 24.8), greater frequency of hospitalization (incidence rate ratio 1.95, 1.75 to 2.17), greater likelihood of compulsory admission (OR 2.73, 2.34 to 3.19) and an increased likelihood of prescription of antipsychotics (2.03, 1.75 to 2.35) or non-antipsychotic mood stabilizers (2.07, 1.77 to 2.41).</td></tr>
</table>

<table>
<tr><th colspan="4">Castro et al. 2015 [39]</th></tr>
<tr><th>Context</th><th>Population</th><th>Data</th><th>Volume</th></tr>
<tr><td>Bipolar disorder</td><td>International Cohort Collection for Bipolar Disorder (Massachusetts General Hospital) patients “spanning more than 20 years”</td><td>EHRs</td><td>4.2 million patients</td></tr>
<tr><td colspan="4">Goal: To diagnose bipolar disorder in EHRs.</td></tr>
<tr><td colspan="4">Method: Manual choice of terms, term detection taking negation into account, LASSO-logistic regression based on those terms. Also rule-based classification for patients with no psychiatric clinical notes. Platform not mentioned.</td></tr>
</table>

**Results:** The PPV of NLP-defined BD was 0.85. A coded classification based on strict filtering achieved a PPV of 0.79, but BD classifications based on less stringent criteria performed less well. None of the EHR-classified controls was given a diagnosis of BD on direct interview (PPV = 1.0). For most subphenotypes, PPVs exceeded 0.80. The EHR-based classifications were used to accrue 4,500 BD cases and 5,000 controls for genetic analyses.

**Shiner et al. 2013 [64]**			
**Context**	**Population**	**Data**	**Volume**
PTSD	Newly enrolled patients in six Veterans' Health Administration (VHA) outpatient PTSD clinics in New England	Clinical notes and administrative data	84,561 clinical notes for 1,924 patients
**Goal:** To describe the application of automated coding to psychotherapy notes. To contribute to measurement of implementation of EBP over time and at multiple levels.			
**Method:** CUIs UMLS extraction (cTAKES). To extract CUIs they are combined with nouns and two classifiers are used: MaxEnt and conditional random fields. Stata software used.			
**Results:** 6.3% of our study population received at least one session of EBP (CPT or PE) during the initial 6 months of treatment.			

**Sohn et al. 2011 [117]**			
**Context**	**Population**	**Data**	**Volume**
Drug side-effects	Patients of psychiatry and psychology department at Mayo Clinic.	Clinical notes	237 patients
**Goal:** To extract physician-asserted drug side effects from clinical narratives, and to associate this effect to one drug. Compare a rule-based system to a machine learning system for extracting side-effects and causative drugs.			
**Method:** CUIs UMLS extraction (cTAKES). Tokenization, lemmatization, chunking, NER. Manually written regular expressions (given in the paper) for detecting drug side effects. As these are not sufficient, training of a C4.5 classifier and finally union of the two results. Apache UIMA platform used.			
**Results:** The rule-based system had a F-score of 0.80, compared to the hybrid system F-score en 0.75. The hybrid system covered more side-effects and causative drug pairs than individual extraction.			

**Perlis et al. 2012 [55]**			
**Context**	**Population**	**Data**	**Volume**
Treatment-resistant depression	Patients of Massachusetts General Hospital (MGH) and Brigham and Women's Hospital (BWH) with diagnosis of isolated major depressive disorders	EMRs, socio-demographic data, biling codes, laboratory results, problem lists, medications, vital signs and narrative notes.	5,198 patients
**Goal:** To classify notes from patients with diagnosis of major depressive disorder as treatment resistant depression or not. To compare NLP classification to the one obtained with billing data (ICD-9 codes) alone and to clinical gold standard (clinician expertise)			
**Method:** Three approaches: billing data only, narrative terms about depression, all data. Term list obtained manually. Using logistic regressuib to obtain three models. i2b2 platform used.			
**Results:** Best performance by NLP model with a precision of 0.78 and a recall of 0.42 for the depressed state, and a precision of 0.86 and a recall of 0.37 for those being well.			

Yu 2011 [118]			
**Context**	**Population**	**Data**	**Volume**
Negative life event classification	Users of 2 psychiatric websites: John Tung Foundation and PsychPark (a virtual psychiatric clinic, maintained by a group of volunteer professionals belonging to the Taiwan Association of Mental Health Informatics)	Forum posts and self-assessment questionnaires	5,000 unlabeled posts and 1,762 labeled records
**Goal:** To retrieve and classify negative life events			
**Method:** First association rules are mined to obtain frequent word sets. Then an embedding is built in which association language patterns of arbitrary length are defined. This allows similarity measure as the inverse of Kullback-Leibler distance. Classification is performed with SVMs, C4.5, Naïve Bayes and tree-augmented Naïve Bayes. Besides bag-of-word and association rules, also hyperonyms/hyponyms from WordNet have been used. Weka platform used.			
**Results:** Best results obtained by SVMs: accuracy of 0.82 with bag-of-words, word patterns and unsupervised Web-based expansion.			
